# Phosphorothioate antisense oligonucleotide induced innate immune activation is attenuated by tryptophan oxidation products

**DOI:** 10.1093/nar/gkag311

**Published:** 2026-04-13

**Authors:** Julia Pytte, Lesley Saldana, Wei Zhang, Konstantina Skourti-Stathaki, Stanley T Crooke

**Affiliations:** n-Lorem Foundation, Carlsbad, CA 92010, United States; n-Lorem Foundation, Carlsbad, CA 92010, United States; n-Lorem Foundation, Carlsbad, CA 92010, United States; n-Lorem Foundation, Carlsbad, CA 92010, United States; n-Lorem Foundation, Carlsbad, CA 92010, United States

## Abstract

Phosphorothioate antisense oligonucleotides (PS ASOs) are a clinically validated therapeutic modality, yet their capacity to activate innate immunity through Toll-Like Receptors, specifically Toll-Like Receptor 9 (TLR9), remains a challenge. While the molecular mechanisms governing TLR9 activation, including PS content, CpG motifs, 2’ modifications, sequence composition, and protein interactions, are well defined, far less is understood about the mechanisms that resolve these responses. Here, we investigate how PS ASO-mediated innate immune activation interfaces with tryptophan metabolizing enzymes, indoleamine 2,3-dioxygenase 1 (IDO1) and interleukin-4–induced-1 (IL4I1), which generate immunomodulatory tryptophan oxidation products. Across lymphoid and myeloid cell systems, we demonstrate that PS ASOs differing in TLR9 agonist potency and kinetics elicit differential induction of IDO1/IL4I1, accompanied by proportional changes in downstream metabolites. Complementary IDO1 knockdown and IL4I1 overexpression experiments alter TLR9-dependent signaling, indicating that these enzymes actively constrain PS ASO-driven immune activation. Finally, exogenous kynurenine and indole pathway metabolites attenuate PS ASO-induced signaling, supporting their role as negative-feedback regulators. Together, these findings solidify a mechanistic link between PS ASO innate immune activation and tryptophan catabolism, revealing enzymatic and metabolite feedback mechanisms that attenuate innate immune signaling, which may be leveraged to enhance the tolerability of immunostimulatory PS ASOs.

## Graphical Abstract

**Graphical Abstract:**
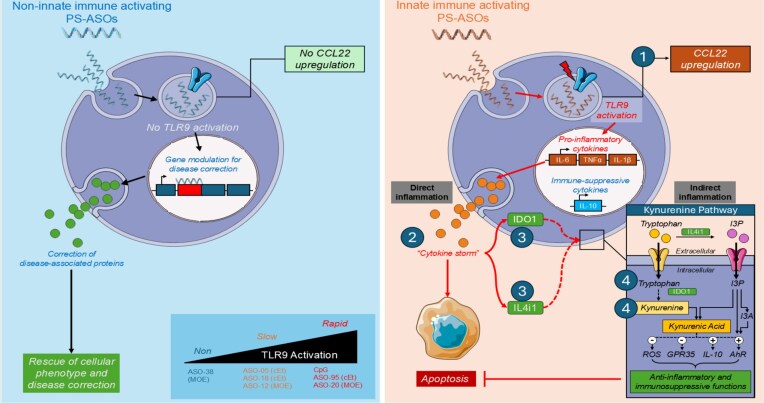

## Introduction

Antisense technology is now a fully validated, broadly enabling drug discovery platform that continues to progress. Recent advances include new pharmacodynamic mechanisms, a more detailed understanding of cell uptake and sub-cellular distribution, conjugates to target PS ASOs to specific organs or cells, a detailed understanding of the molecular mechanism of cytotoxicity for PS ASOs (and approaches to avoid cytotoxicity), and new chemical modifications, all of which have been extensively reviewed [1, 2, 3]. New insights into the molecular mechanisms by which PS ASOs induce innate immunity have been reported, as PS ASO-induced innate immunity can result in adverse events in patients. Recent data demonstrate that PS ASOs with different 2’ modifications activate innate immunity via TLR9 [4], that PS ASO binding proteins modulate TLR9 activation [5], and that PS ASOs engage both CpG and non-CpG sites in a TLR9 dimer [6]. Accordingly, PS ASOs can behave as full or partial TLR9 agonists, competitive antagonists, or cooperatively enhance TLR9 activation when combined with partial agonists [6]. Additionally, previous studies have shown that different PS ASOs can display different kinetics of innate immune activation [5]. Despite these improvements in understanding how PS ASOs initiate innate immune activation, and although the negative-feedback architecture of MyD88-dependent TLR signaling is broadly understood [7, 8], how these regulatory circuits operate in the specific context of PS ASO-driven TLR9 activation, including their kinetics relative to the strength and duration of the initial stimulus, remains underexplored.

Parallel to these advances, interest has grown in the kynurenine pathway (KP) and related indole-oxidative pathways as immunoregulatory systems that reshape inflammatory signaling (Supplementary Fig. S1) [9]. While plasma Trp, L-kynurenine (Kyn), and Kyn/Trp ratio have served as clinical biomarkers for innate immune activation [10–12, 13], focus has shifted to the identification of druggable targets in the pathway and the therapeutic potential of anti-inflammatory co-therapy with Kyn or Kynurenic acid (KYNA) to improve patient outcomes [14]. Given that Kyn is produced via the activity of the enzyme indoleamine 2,3-dioxygenase (IDO1) and is an agonist of the immunomodulatory aryl hydrocarbon receptor (AhR) pathway, it has been postulated that Kyn could represent a potential anti-immunogenic co-therapy [15]. Additionally, it has also been reported that interleukin 4 induced 1 (IL4I1), an L-amino acid oxidase, can metabolize Trp to indole-3-pyruvic acid (I3P) and indole-3-acetaldehyde (I3A) and terminate innate immune activation [16, 17]. As such, we sought to explore the role of these key players in response to both immunogenic and non-immune stimulatory PS ASOs and determine the extent of their activation. These observations raise an essential question: how strongly do PS-ASO-induced innate responses engage tryptophan-oxidative pathways, and do these pathways modulate TLR9 signaling? Given the wide variation in PS-ASO TLR9 activation, clarifying the induction of IDO1/IL4I1, changes in tryptophan metabolites, and the effects of manipulating these pathways is critical for understanding how cells regulate innate signaling.

Here, we address this gap using a panel of PS ASOs with well-characterized TLR9 agonist properties, including both CpG and non-CpG sequences, and “fast” and “slow” innate immune activators tested across complementary lymphoid (BJAB) and myeloid (THP1-TLR9) cell lines [4, 5, 6]. The Trp oxidation enzymes IDO1 and IL4I1, which are broadly inducible regulatory signals, are upregulated by PS ASO-mediated innate immune activation. Notably, the kinetics and scale of the induction of these enzymes correlate with the kinetics and scale of innate immune stimulation induced by PS ASOs. Genetic manipulation of the Trp oxidation enzymes IDO1/IL4I1 alters PS ASO innate immune activation, as silencing of IDO1 increases PS ASO innate immune activation and overexpression of IL4I1 suppresses PS ASO innate immune activation. In cells that express both IDO1 and IL4I1, tryptophan is consumed, and levels of N-formyl-L-Kynurenine (N-fkyn), Kyn, 3-hydroxyl-L-Kynurenine (3HK), 3-Hydroxyanthranilic Acid (3HAA), Formylanthranilic Acid (FAA), and Anthranilic Acid (AA) are elevated. The addition of metabolites from both the kynurenine and indole pathways suppresses PS ASO-mediated innate immune responses in myeloid cells that express both IDO1 and IL4I1, and lymphoid cells that express only IL4I1. Moreover, we have demonstrated that tryptophan oxidation products administered to cells can suppress PS ASO-induced innate immune activation, raising the possibility that formulating PS ASOs with tryptophan oxidation products might suppress PS ASO-induced innate immune activation in patients. Together, these studies delineate a mechanistic framework in which tryptophan catabolism acts as an inducible negative-feedback module that shapes PS-ASO-induced innate immunity, with implications for designing PS ASOs with improved tolerability and for identifying metabolic adjuncts that mitigate immunostimulatory effects

## Material and methods

### Materials

Primer-probe sets (Supplementary Table S1), siRNAs (Supplementary Table S2), ELISAs (Supplementary Table S3), and antibodies (Supplementary Table S4) are listed in the Supplementary Materials. All synthetic oligonucleotides were designed and synthesized at Ionis Pharmaceuticals (Carlsbad, CA). PS ASOs were diluted in phosphate-buffered saline (PBS) for *in vitro* usage.

### Cell culture

THP1-Dual hTLR9 cells (InvivoGen, thpd-htlr9) were grown according to the manufacturer’s instructions in a maintenance medium of Roswell Park Memorial Institute (RPMI) 1640 Medium + L-Glutamine (Gibco, 11875093) supplemented with 10% (v/v) heat-inactivated fetal bovine serum (Gibco, A5256801), 25 mM HEPES (Gibco, 15630080), 100 U/mL penicillin and streptomycin solution (Cytiva, SV30010), 100 µg/mL normocin (InvivoGen, ant-nr-05), under standard conditions of 37°C, 5% CO_2_. Selection antibiotics: 100 µg/mL Zeocin (InvivoGen, ant-zn-05), 10 µg/mL Blasticidin (InvivoGen, ant-bl-05), and 1 µg/mL Puromycin (InvivoGen, ant-pr-1) were applied every second passage.

BJAB cells (DSMZ, ACC 757) & Mono-Mac-1 cells (DSMZ, ACC 252) were obtained from the DSMZ cell repository (Braunschweig, Germany), and KARPAS-1718 cells (Sigma-Aldrich, 08072401) were obtained from Sigma Aldrich. All lines were maintained according to the manufacturer’s instructions in RPMI 1640 Medium + L-Glutamine and were supplemented with 20% or 10% (v/v) heat-inactivated fetal bovine serum and 100 U/mL penicillin and streptomycin solution.

### ASO treatment

Prior to treatment, cell lines were washed in RPMI medium at 150 g x 10 min and seeded in a V-bottom 96-well plate. THP1-TLR9 cells were seeded at a density of 100 000 cells per well in 50 µL, and BJAB cells were seeded at a density of 50 000 cells per well in 50 µL. When large volumes of supernatant were required for metabolite analysis, THP1-TLR9 cells were seeded in six-well plates at a density of 3 × 10^6^ cells per well in 1.5 mL. Cell lines were incubated at 37°C, 5% CO_2_, with 50 µL or 1.5 mL of the indicated PS ASO for free uptake. For pulse treatment, after 2 h of incubation, cells were collected into 15-mL Falcon tubes, or 96-well v-bottom plates were centrifuged directly at 350 g x 5 min, and the PS ASO treatment was removed. For recovery, 200 µL or 4 mL of recovery media (RMPI 1640 + 10% heat-inactivated FBS) was dispensed into the 96-well plate or six-well plate, respectively, and subsequently returned to the incubator. RNA and/or supernatant collection occurred between 8 and 72 h post-treatment. For continuous treatment, cell lines were incubated with the indicated PS ASO for 2–24 h at 37°C, 5% CO_2_, with RNA collected immediately.

### Tryptophan metabolite treatment

To characterize the immunosuppressive effect of tryptophan metabolites, relevant cell lines were dosed with either Kyn (Sigma-Aldrich, K8625), KYNA (Sigma-Aldrich, K3375), 3HAA (Sigma-Aldrich, 148776), and I3P (Sigma-Aldrich, I7017), at various concentrations (0.08 uM–1.5mM) in a dose-dependent study. Cell lines were washed in serum-free RPMI at 150 g x 10 min prior to seeding into a 96-well plate at a density of 50 000 cells per well in 50 µL for BJAB cells, and 100 000 cells per well in 50 µL for THP1-TLR9 cells. Cell lines were incubated at 37°C, 5% CO₂, with 50 µL of the indicated excipient in serum-free RPMI for 4 h. After excipient pre-treatment, cell lines were dosed with 50 µL of PS-ASO, to a final concentration of 0.8 µM in recovery media (RPMI + 10% Heat inactivated FBS) and subsequently incubated with ASO at 37°C, 5% CO₂. THP1-TLR9 supernatants were collected at 4 h for downstream IRF and NF-kB activity analysis. Lysates were collected at 8 h for BJAB cells and 24 h for THP1-TLR9 cells for downstream RNA analysis.

### RNA isolation and extraction

Following PS ASO treatment, v-bottom plates were centrifuged at 1000 g x 5 min to pellet cells. After media removal, cells were lysed in Guanidine Isothiocyanate Solution (GITC) (Invitrogen; 15577-018). RNA was isolated using 384-well glass fiber filter plates (Pall, 5072), and washed twice with RNA wash buffer (60 mM Potassium Acetate (Thermo Scientific Chemicals, J62817AK), 10 mM Tris-HCL (Invitrogen, 15568025), 60% (v/v) Ethanol (Fisher Scientific, BP2818-4)) prior to a 20 min DNAse treatment (Invitrogen, 18047019). After DNase treatment, RNA was washed twice with dilute GITC buffer (75% (v/v) ddH_2_O, 25% (v/v) GITC buffer), twice with RNA wash buffer, and eluted using ddH_2_O. RNA quantity and purity were determined using a NanoDrop Lite (Thermo Scientific, NDLPLUSGL).

### qRT-PCR

qRT-PCR was performed in triplicate using TaqMan primer-probe sets and AgPath-ID™ One-Step RT-PCR Reagents (Thermo Scientific, #4387391) according to the manufacturer’s instructions. Briefly, ∼50 ng RNA in 4 µL was mixed with 0.25 µL primer-probe sets containing forward and reverse primers (9 µM of each) and fluorescently labeled probe (3.75 µM), 5 µL 2x RT-PCR Buffer, 0.4 µL RT-PCR Enzyme Mix, and 0.35 µL nuclease-free ddH_2_O in a 10 µL reaction. RNA was reverse transcribed at 45°C for 10 min, inactivated and denatured at 95°C for 10 min, and amplified for 40 cycles at 95°C for 15 s and 60°C for 45 s with data collected at the end of each cycle on a QuantStudio™ 7 Pro Real-Time PCR System (Applied Biosystems, A43183). Results were analyzed by the relative quantity (ddCt) method. Where relevant, mRNA levels were normalized to the amount of total RNA present in each reaction as determined for duplicate RNA samples using the Ribogreen Assay (Invitrogen, R11490).

### Protein extraction and western blot analysis

Cell pellets were lysed in RIPA Buffer (Thermo Scientific, 89901) at 4°C for 30 min, and sonicated for 1 s x 3 pulses at 30% Amplitude on a SONICS Vibra Cell Ultrasonic Processor (Sonics & Materials Inc., VCX 600). Proteins were clarified by centrifugation at 14 000 g x 15 min at 4°C, with concentration determined using a Pierce BCA protein Assay Kit (Thermo Fisher, 23227). Clarified protein samples were prepared with Laemmli Sample Buffer (BioRad, 1610747) and 2-Mercaptoethanol (BioRad, 1610710), and ∼15 ug of each sample was separated on a 4–20% Mini-Protean TGX Stain-free Precast Gel (BioRad, 4568094). Separated protein samples were transferred onto 0.2 µm polyvinylidene difluoride (PVDF) membranes (BioRad, 1704156) using a Trans-Blot® Turbo™ Transfer System (BioRad, 1704150). The membranes were blocked at room temperature for 1 h in 5% non-fat dry milk in 1x Tris Buffer Saline (BioRad, 1706435) + 0.1% Tween (Biorad, 1706531) (TBST). Membranes were probed with primary antibodies at 4°C overnight. After three 5 min washes with 1x TBST, the membranes were incubated with appropriate HRP-conjugated secondary antibodies (1:5000) at room temperature for 1 hr. Following three 5 min washes with 1x TBST, the membranes were visualized using Immobilon Forte Western HRP Substrate (Millipore, WBKLS0500) on a ChemiDoc Imaging System (BioRad, 12003153).

### siRNA treatment

Around 100 µL of 1 × 10^6^ THP1-TLR9 cells and 1 µM of siRNA were added to a BTX high-throughput electroporation plate. The cells were electroporated at 260 V for 6 ms using an ECM 830 high-throughput electroporation system. At 24 h after siRNA electroporation, cells were reseeded for PS ASO treatment.

### Overexpression treatment

BJAB cells were washed in reduced serum Opti-MEM (Fisher, 31-985-070) at 150 g x 10 min, and seeded in six-well plates at 2 × 10^6^ cells per well in 2 mL Opti-MEM. 2.5 µg of IL4I1 (NM_152899) Human Tagged ORF clone (OriGene Tech, RC210310) was transfected into cells using Lipofectamine 3000 (ThermoFisher, L3000015) according to the manufacturer’s instructions. Cells were incubated at 37°C, 5% CO_2_ for 8 h before collection into 15-mL Falcon tubes and centrifugation at 150 g x 10 min to remove treatment. Cells were re-seeded in 2 mL of recovery media (RPMI + 10% heat inactivated FBS) and returned to the incubator for 16 h. After 16 h of recovery, CpG ASO was dispensed to designated wells to a final concentration of 3.2 µM. Lysates for downstream RNA were collected at 24 h.

### Lumit and ELISA assay

For the analysis of secreted cytokines and metabolites, 150 µL of culture media was transferred to a separate assay plate and stored at −80°C for future analysis during RNA collection. Lumit assays (Promega) for quantification of secreted TNF-α, IL-1β, IL-6, and IL-10 were conducted according to the manufacturer’s instructions using 20 µL of supernatant in white 384-well plates. Luminescence was measured using the GloMax Discover Microplate Reader (Promega, GM3000). ELISA assays (Immunsol) for quantification of secreted Kyn and Trp were conducted according to the manufacturer’s instructions. Absorbance was measured using the GloMax Discover Microplate Reader (Promega).

### NF-kβ and ISG reporter assays

THP1-Dual hTLR9 cells (InvivoGen) overexpress the human TLR9 gene and are engineered with two secreted reporters. *Lucia* luciferase under an ISG54 (interferon-stimulated gene) promoter in conjunction with five interferon-stimulated response elements, and secreted embryonic alkaline phosphatase reporter under an IFN-β minimal promoter fused to NF-kβ and c-Rel binding site. NF-kB activity was quantified using the Quanti-Blue kit (rep-qbs, Invivogen) according to the manufacturer’s instructions. Briefly, 20 µL of thawed supernatant was transferred to a clear 96-well plate and combined with 180 µL of Quanti-Blue solution (InvivoGen). Samples were incubated for 1 h at 37°C prior to absorbance being measured using the GloMax Discover Microplate Reader. ISG activity was quantified using the Quanti-Luc 4 Lucia/Gaussia kit (Invivogen, rep-qlc4lg1) according to the manufacturer’s instructions. Briefly, 20 µL of thawed supernatant was transferred to a white opaque 96-well plate and mixed with 50 µL of Quanti-Luc 4 Reagent. Luminescence was recorded immediately using the GloMax Discover Microplate Reader with 0.1 s integration time.

### Targeted metabolomics analysis of the kynurenine pathway

#### Sample preparation

THP1-TLR9 cells were seeded in six-well plates at a density of 3 × 10^6^ cells per well in 1.5 mL. Cell lines were incubated at 37°C, 5% CO_2_ with 1.5 mL of the indicated PS ASO for 2 h, to a final concentration of 8 µM. Cells were collected into 15-mL Falcon tubes, centrifuged at 350 g x 5 min, and the PS ASO treatment was removed. Cells were resuspended in 4 mL of recovery media (RPMI 1640 + 10% heat-inactivated FBS), dispensed into a six-well plate, and incubated for 48 h. Supernatants were collected and stored at −80°C until analysis.

Quantitative metabolomics was performed by MtoZ Biolabs (Boston, MA, USA). Briefly, supernatants were thawed, vortexed, and 50 μL was mixed with 250 μL 20% (v/v) acetonitrile/methanol, vortexed for 3 min, and centrifuged at 12 000 rpm for 10 min at 4°C. 250 μL of clarified supernatant was chilled at −20°C for 30 min, re-centrifuged, and 180 μL of supernatant was transferred to a protein precipitation plate for further LC-MS analysis.

For carbonyl-acid derivatization, 30 μL of supernatant extract was combined with 5 μL 200mM 3-nitrophenylhydrazine (3-NPH) in 75% methanol and 5 μL of 150 mM EDC solution with 4.5% pyridine in methanol in 1.5 mL tubes. The mixture was incubated at 30°C for 60 min, diluted with 260 μL acetonitrile, and chilled at −20°C for 30 min before 2 μL was injected into the LC-MS system for further analysis.

#### LC-ESI-MS/MS instrument parameters

Analysis was performed on ExionLC AD UPLC coupled to a QTRAP 6500 + mass spectrometer with an ESI Turbo V (SCIEX). The column oven was set to 40°C with an injection volume of 2 μL. Metabolites were analyzed by reverse-phase chromatography on a HSS T3 C18 column (Acquity UPLC HSS T3, 100 mm × 2.1 mm, 1.8 μm) at a flow rate of 0.35 mL/min. The mobile phase consisted of 0.05% formic acid in water (A) and 0.05% formic acid in acetonitrile (B). The gradient conditions were as follows: 5% B at 0 min, 95% B at 8 min, and held at 95% B until 12.5min, 5% B at 12.6 min, and held at 5% B until 15 min. Each run lasted 15 min. In addition, derivatized samples were separated using the same parameters, except the gradient conditions were as follows: 30% B at 0 min, held to 0.5 min, 95% B at 3.5 min, held to 4.9 min, 30% B at 5 min, held to 6 min. Each run lasted 6 min. Polar metabolites were analyzed by hydrophilic interaction chromatography on a BEH Amide column (Acquity UPLC BEH Amide, 100 × 2.1 mm, 1.7 μm; Waters) at a flow rate of 0.40 mL/min. The mobile phase consisted of 10 mM Ammonium acetate and 0.3% ammonium hydroxide in water (A), and acetonitrile/water, 90:10 (v/v). The gradient conditions were as follows: 95% B at 0 min, held to 1.2 min, 70% Bat 8 min, 50% B at 9.0 min, held to 11.0 min, and equilibrated at 95% B from 11.1 to 15 min. Each run was 15 min.

#### Mass spectrometry and quantification

ESI–MRM mass spectrometry was performed in positive and negative modes under Analyst 1.6.3 control (SCIEX) on a QTRAP 6500 + triple quadrupole-linear ion trap equipped with an ESI Turbo V source (550°C, +5500/−4500 V, curtain gas 35 psi). Data were acquired in scheduled MRM with compound-specific retention-time windows; declustering potentials and collision energies were optimized per transition using authentic standards. Raw .wiff/.wiff.scan files were processed in MultiQuant 3.0.3 (SCIEX) for peak integration and quantification using external, matrix-matched calibration with isotopically labeled internal standards where available.

### Statistical analysis

Statistical analysis was conducted using GraphPad Prism Software (v10.4.0). Whenever relevant, the statistical significance is determined by ANOVA followed by Dunnett’s post-hoc test. When correlations between various endpoints were obtained, R values were reported using Spearman’s rank correlation analysis. Linear regression with normalized response and variable slope was utilized to calculate IC50. A *P*-value of less than 0.05 was considered statistically significant for all analyses.

## Results

### PS ASO innate immunogenicity varies between different cell systems

**Figure 1.**
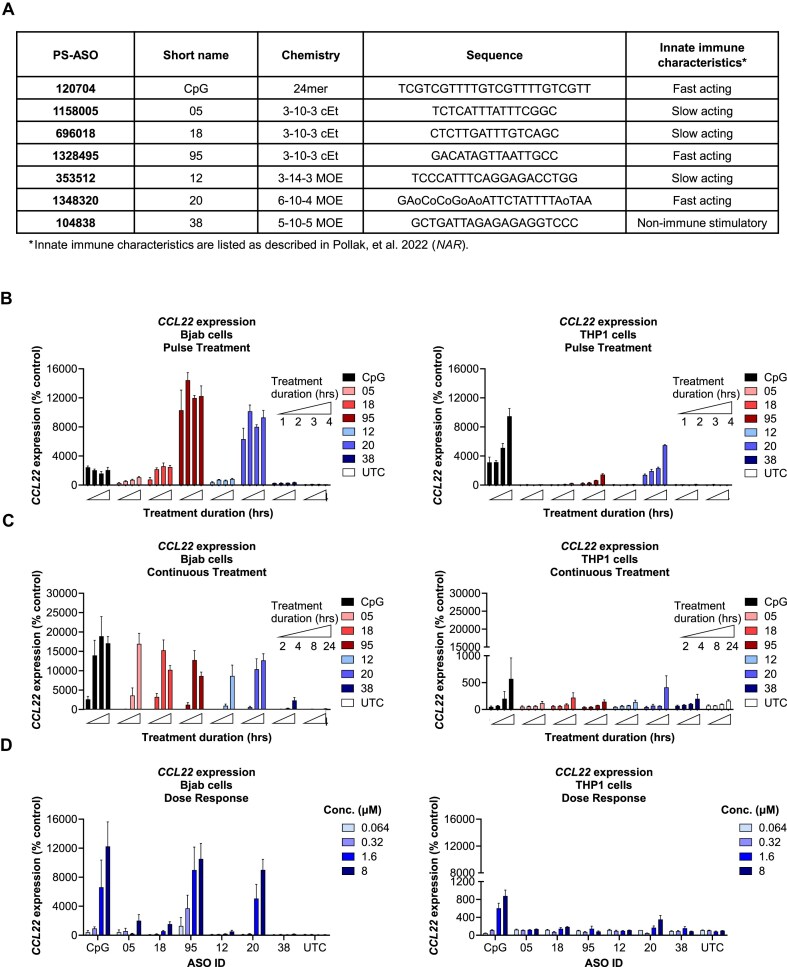
PS ASO innate immunogenicity varies with cell system, treatment duration, and concentration. (**A**) Model PS-ASOs used for this study. “o”indicates a phosphodiester linkage; all other linkages are phosphonothioate. Constrained Ethyl (cEt) and MOE 2’ modifications are indicated for each PS-ASO. Fast-acting or slow-acting TLR9 agonism is listed as defined by Pollak et al. 2022 ([5], [6]). (**B**) Pulse treatment in the BJAB cell line (left) and the THP1-TLR9 cell line (right). Relative qRT-PCR levels of *CCL22* mRNA following a 1, 2, 3, or 4 h incubation with 1.6 µM of the indicated PS ASOs in serum-free RPMI media. RNA lysates were collected 24 h following treatment. (**C**) Continuous treatment in the BJAB cell line (left) and the THP1-TLR9 cell line (right). Relative qRT-PCR levels of *CCL22* following a 2-, 4-, 8-, or 24-h incubation with 1.6 µM of indicated PS ASOs in serum-free RPMI media. RNA lysates were collected immediately following treatment. (**D**) Dose response in BJAB cell line (left) or THP1-TLR9 cell line (right). Cells were treated for 2 h with varying concentrations (0.064, 0.32, 1.6, or 8 µM) of PS ASOs. RNA lysates were collected 24 h after treatment. All experiments were reproduced in at least three independent biological experiments, with multiple replicates per experiment.

We first established a reliable cell model and assay to study cellular pathways responsible for suppression of PS ASO-mediated innate immune response. We selected a panel of PS ASOs previously characterized by Pollak et al. (2022) as full or partial agonists and as “fast” or “slow” TLR9 agonists, defined as the differences in the kinetics of TLR9-dependent innate immune activation [5, 6], and examined these responses in both a lymphoid and myeloid cell system (Fig. 1A). As BJAB cells are a validated predictive model of PS ASO innate immunogenicity, due to factors such as limited heterogeneity, stable high TLR9 expression, and reproducible NF-κβ-dependent cytokine/chemokine responses to PS ASOs, they were included as a reference control in all experiments [5, 18–23, 24]. As lymphoid cells lack IDO1 [25], a human monocyte-derived cell line that overexpresses the human TLR9 gene, THP1-TLR9 cells, was used to study IDO1-mediated innate immune suppression. In addition, the lymphoid line KARPAS1718 and monocytic line MONOMAC-1 were included due to their high *TLR9* and *CCL22* expression, and selectivity for either *IDO1* or *IL4I1* expression (Supplementary Table S5.). We initially characterized differences between these cell systems in response to PS ASO immune activation, with the specific goal of determining whether a brief “pulse” of PS-ASO exposure (1–4 h) followed by washout or recovery is sufficient to fully commit cells to a TLR9-driven response, or whether continuous treatment (2–24 h) is required (Fig. 1B, C). Due to the inability of the Karpas-1718 cells and MonoMac-1 cells to register the broad dynamic range of PS-ASOs’ innate immunogenicity, in all the conditions tested, we pursued experiments in the validated BJAB cell line and THP1-TLR9 cell line (Supplementary Fig. 2A, B).

A side-by-side comparison between the BJAB and THP1-TLR9 cell systems demonstrates distinct differences in responses to immune stimulation in response to 1–4 h pulse PS ASO treatment, with both CpG and Non-CpG PS ASOs (Fig. 1B). TLR9 activation was measured via readout of C-C motif chemokine ligand 22 (CCL22) mRNA expression, an established system to predict PS ASO immune responses in humans [20]. In the BJAB cell system, both fast (CpG, ASO-95, and ASO-20) and slow immune stimulators (ASO-05, ASO-12, and ASO-18) caused an increase in TLR9 activation, regardless of the duration with which the cells were treated with PS ASOs. This, however, was not observed in the THP1-TLR9 cells, as only fast immune stimulators, as well as ASO 18, caused an increase in TLR9 activation after pulse treatment. Also, the observation that the CpG ASO was more potent in THP1-TLR9 cells than in the BJAB cells. Interestingly, this pattern of TLR9 stimulation in THP1-TLR9 cells is very similar to TLR9 activation observed with the same PS ASOs in HEK-293-TLR9 cells [5]. Overall, we observe that a 2-h pulse treatment allows sufficient PS-ASO to be internalized, triggering TLR9 and rank ordering the PS-ASOs across a broad dynamic range, and that extending treatment time beyond 2 h had minimal impact on the effects of PS-ASOs and CpG-containing oligonucleotides on the innate immune response. This is unsurprising, considering the kinetics of cellular uptake and sub-cellular distribution and the fact that TLR9 is localized to late endosomes [26, 27].

In addition to the pulse treatment, PS ASOs were applied continuously for 2, 4, 8, or 24 h, with TLR9 activation measured immediately after treatment. The earliest response to a PS ASO was observed in the BJAB cell system, with the CpG ASO upregulating *CCL22* after only 2 h of exposure (Fig. 1C). ASO-20, ASO-95, and ASO-18 all elicited a weak response at 4 h, followed by a stronger response at 8–24 h. ASO-05 and ASO-12 elicited a response at 8–24 h. These data confirm previous reports that PS ASOs vary in their kinetics and strength of innate immune activation, regardless of chemical modifications. For the THP1-TLR9 cells, the CpG ASO was the only PS ASO that elicited a response at 8 h, whereas ASO-20 and ASO-95 elicited a response at 24 h. Comparing the pulse and continuous treatment, we observe better discrimination between PS-ASOs with regard to both the magnitude and kinetics of innate immune activation using the pulse treatment. Consequently, we used 2-h pulse treatments for most experiments.

To further understand how PS ASOs behave in the two-cell systems, a dose-response study was conducted (Fig. 1D). As expected, increasing concentrations of PS ASOs increased TLR9 activation, with a peak in response observed between 1.6 and 8 µM for all PS ASOs except ASO-18, where peak response is observed at 200 µM (Supplementary Fig. 2C). Due to significant cytotoxicity observed between the 40 and 200 µM range dose, we limited subsequent experiments to doses of 8 µM and lower (Supplementary Fig. 2D). A peak in TLR9 response between 1.6 and 8 µM was also observed in the THP1-TLR9 cells for CpG, ASO-18, and ASO-20; however, little to no response was observed for other PS ASOs in the THP1-TLR9 cell system. Our data have established that while the two cell systems respond differently to PS ASOs, there is an immediate response to innate immune activation in both cell models. Interestingly, in THP1-TLR9 cells, the CpG ASO was more potent after pulse treatment than in BJAB. This tends to reduce the likelihood that differences in the number or coupling of TLR9 receptors account for the differences observed in responses of the two cell lines. Though the precise mechanism that accounts for the differences observed between the two cell lines remains elusive, our data confirm that different cell lines may respond to innate stimulants differently, encouraging caution in extrapolating data from a single cell line too generically.

### Kinetics of cytokines released in response to PS ASOs vary according to sequence and chemistry

To determine whether the cell-specific differences in the ability to detect immunogenic responses of PS ASOs resulted from alternative downstream TLR9-mediated cytokine expression, we examined the expression profiles of key secreted pro-inflammatory cytokines, tumor necrosis factor alpha (TNF-α), Interleukin-1 beta (IL-1β) and interleukin-6 (IL-6), all of which are significantly altered in human peripheral blood mononuclear cells treated with PS ASOs [24]. To determine if myeloid differentiation of primary response gene 88 (*Myd88*) plays a role in PS ASO caused innate immune activation, we also examined the release of the anti-inflammatory IL-10 cytokine [5, 28].

**Figure 2.**
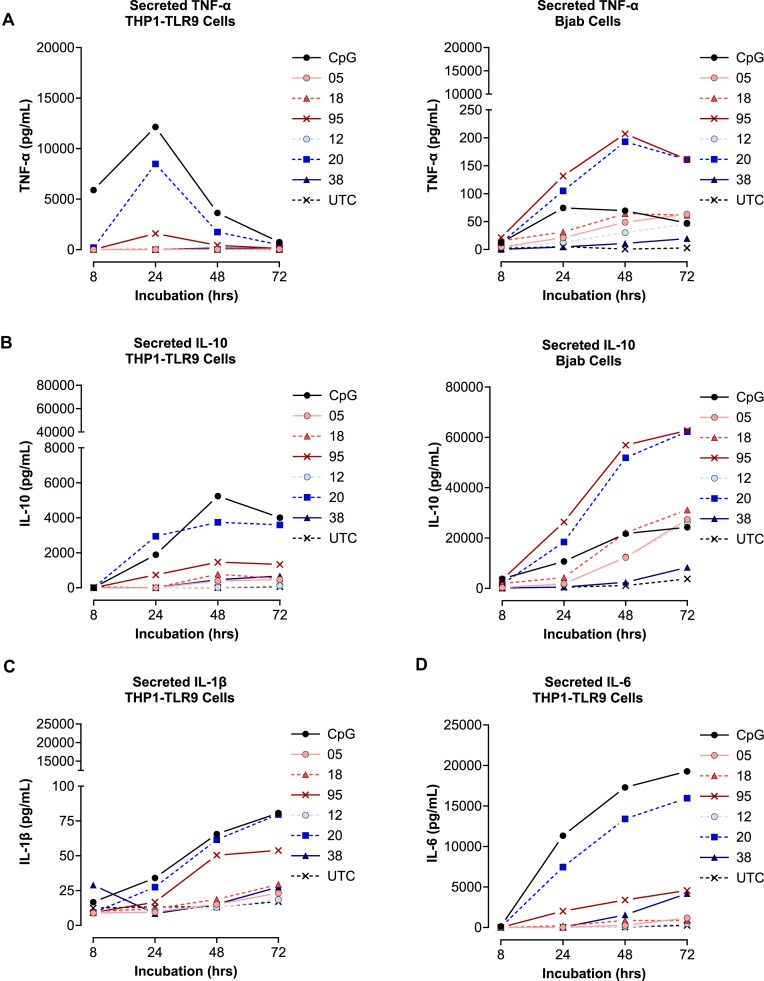
Differential kinetics of cytokine secretion following PS ASO stimulation in BJAB and THP1-TLR9 cells. THP1-TLR9 (left) and BJAB cells (right) were treated with 1.6 μM of the indicated PS ASOs for 2 h, before supernatants were collected for cytokine analysis of TNF-α (**A**) and IL-10 (**B**). THP1-TLR9 cells were also assessed for levels of secreted IL-1β (**C**) and IL-6 (**D**). Values were interpolated to a standard curve of known protein concentration, and are expressed as mean at each time point. All experiments were reproduced in at least three independent biological experiments, with multiple replicates per experiment.

TNF-α in the THP1-TLR9 cell line peaked at 24 h with fast TLR9 agonist PS ASOs, before rapidly declining (Fig. 2A). Levels of TNF-α were negligible in THP1-TLR9 cells treated with slow /partial agonists (ASO-18, ASO-12, and ASO-05), corresponding to low levels of *CCL22* observed previously (Fig. 1B). As expected, TNF-α rapidly increased after treatment with the fast agonists in the BJAB cell line, with the CpG oligo exhibiting maximal activity at 24 h, whereas ASO-20 and ASO-95 exhibited maximal activity at 48 h. Gradual increases in TNF-α were observed with slow agonists, with a plateau occurring at ∼48–72 h. When assessing IL-10 secretion in the THP1-TLR9 cells, fast agonists exhibited a gradual, persistent increase in IL-10, which plateaus at 48 h; however, the magnitude of IL-10 was greatly decreased in ASO-95-treated cells. Interestingly, ASO-38, ASO-18, and ASO-05 demonstrated a limited increase in IL-10 from 48 to 72 h, whereas ASO-12 did not increase IL-10 (Fig. 2B). Both fast and slow agonists cause substantial IL-10 secretion in the BJAB cell line. ASO-95 and ASO-20 show the most dramatic increase in IL-10, whereas the CpG oligo and slow agonists only moderately increase IL-10, a likely counterbalance to the activation of TLR9 via MyD88 signaling. As IL-1β and IL-6 levels were too low to detect in the BJAB cell system, only results for the THP1-TLR9 cell system are shown (Fig. 2C, D) . IL-1β levels increased substantially for fast agonists only, with maximal activity observed at 72 h. IL-6 levels increase rapidly after exposure to fast agonists; however, ASO-95 is only moderately increased.

Considering all the cytokines evaluated, these data show that PS-ASOs segregate into multiple classes based on the extent of innate immune activation and the kinetics of cytokine induction, and that the previously defined fast and slow PS-ASO classes are recapitulated at the level of secreted cytokines. Moreover, the magnitude of TNF-α, IL-6, IL-1β, and IL-10 secretion functionally separates highly inflammatory PS-ASOs from weaker agonists and reveals a clear coupling between pro-inflammatory cytokines and IL-10–mediated feedback in both monocytic and B-cell systems. Serendipitously, we observed morphological changes in THP1-TLR9 monocytes after incubation with the fast agonist PS ASOs, CpG, ASO-20, and ASO-95, but not others (Supplementary Fig. 3). This is not surprising, as it is well known that THP1 cells readily differentiate into macrophages and/or dendritic cells after exposure to strong immune activators such as Lipopolysaccharide (LPS), likely through the release of TNF-α [29]. Overall, these data demonstrate that there is an immediate response to rapid TLR9 agonists that is detectable via secretion of TNF-α, corroborating previous *CCL22* mRNA expression data. Conversely, slow TLR9 agonists remain undetectable in the THP1-TLR9 cell system via TNF-α, IL-1β, and IL-6, also corroborating previous *CCL22* mRNA expression data (Fig. 1).

### PS ASO TLR9 agonists upregulate trp metabolism enzymes IDO1 and IL41

**Figure 3.**
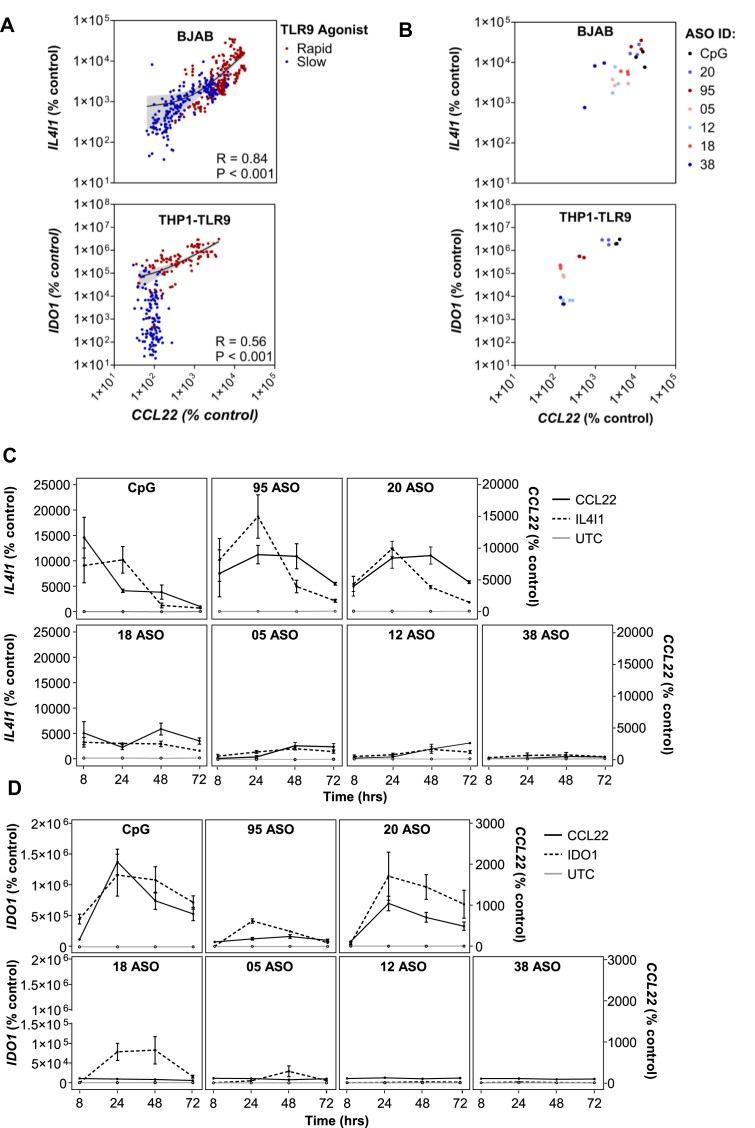
Trp degradation enzymes, *IDO1* and *IL4I1*, are upregulated in immune cells stimulated with PS ASOs. (**A**) Correlation of tryptophan oxidation enzymes and TLR9 activation in BJAB cells (top) and THP1-TLR9 cells (bottom) after treatment with 1.6 µM of the indicated PS ASOs for 2 h. Relative qRT-PCR levels of CCL22 and IL4I1/IDO1 mRNA were measured at 8, 24, 48, and 72 h post-treatment. Spearman’s rank correlation was used to assess the association between CCL22 mRNA and IL4I1/IDO1 mRNA. (**B**) Maximum values observed in each experiment with the indicated PS ASOs for BJAB cells (top) and THP1-TLR9 cells (bottom). (**C**) Kinetics of IL4I1 and CCL22 qRT-PCR levels in BJAB cells, and (**D**) THP1-TLR9 cells after dosing with the indicated PS ASOs. All data are presented as a percentage of UTC control (mRNA expression/Ribogreen/UTC) and expressed as mean ± S.E.M. All experiments were reproduced in at least three independent biological experiments, with multiple replicates per experiment.

As the Kynurenine Pathway (KP) is emerging as a potential modulator of innate immune activation, we examined whether key enzymes involved in the oxidation of Trp to Kyn were altered in response to PS ASO treatment. We observed a positive correlation between TLR9 activation and *IL4I1* expression in the BJAB cell line (Spearman’s *r* = 0. 0.8394, 95% CI: 0.81–0.87, *P* > 0.0001) and *IDO1* expression in the THP1-TLR9 cell line (Spearman’s *r* = 0.5590, 95% CI: 0.46–0.64, *P* > 0.0001; Fig. 3A). As expected, slow TLR9 agonists exhibited lower *IL4I1*/*IDO1* and *CCL22* levels compared to fast agonists, even when only the maximum values of each experiment are considered (Fig. 3B). We also examined the kinetics between mRNA expression of *IL4I1*/*IDO1* and *CCL22* in the two cell lines across multiple time points (Fig. 3C, D). In both cell systems, *IDO1* and *IL4I1* were minimally upregulated after treatment with the non-inflammatory ASO 38. This suggests that well-tolerated, non-inflammatory PS ASOs do not activate TLR9, and do not engage the compensatory KP, aside from ASO 18, which remains an outlier in this experiment. Similar to our previous data, ASO-05 and ASO-12 upregulated *CCL22* and *IL4I1* in the BJAB cell line, but did not do so in the THP1-TLR9 cells, reinforcing that these PS ASOs are not immunogenic in THP1-TLR9 cells. An interesting exception to this is ASO-18, which only appeared to upregulate *CCL22* after 3-4 h of treatment (Fig. 1B), yet demonstrated an increase in *IDO1* when treated for 2 h at the 24 and 48 h time points. Although different PS-ASOs show a wide range of TLR9 activation as previously reported, the rank order of TLR9 activity does not fully predict the magnitude of IDO1/IL4I1 induction, or the kinetics of this induction. This builds evidence that the SAR governing the PS-ASO-mediated innate immune response activation and the SAR governing downstream immunoregulatory tryptophan catabolism are similar, but not identical. Nevertheless, we observed a relatively proportional relationship between levels of TLR9 activation and IDO1/IL4I1 induction in the THP1-TLR9 and BJAB cell lines, respectively.

### Kynurenine induction and TLR9 activation by immunogenic PS-ASOs depend on tryptophan-catabolizing enzymes

**Figure 4.**
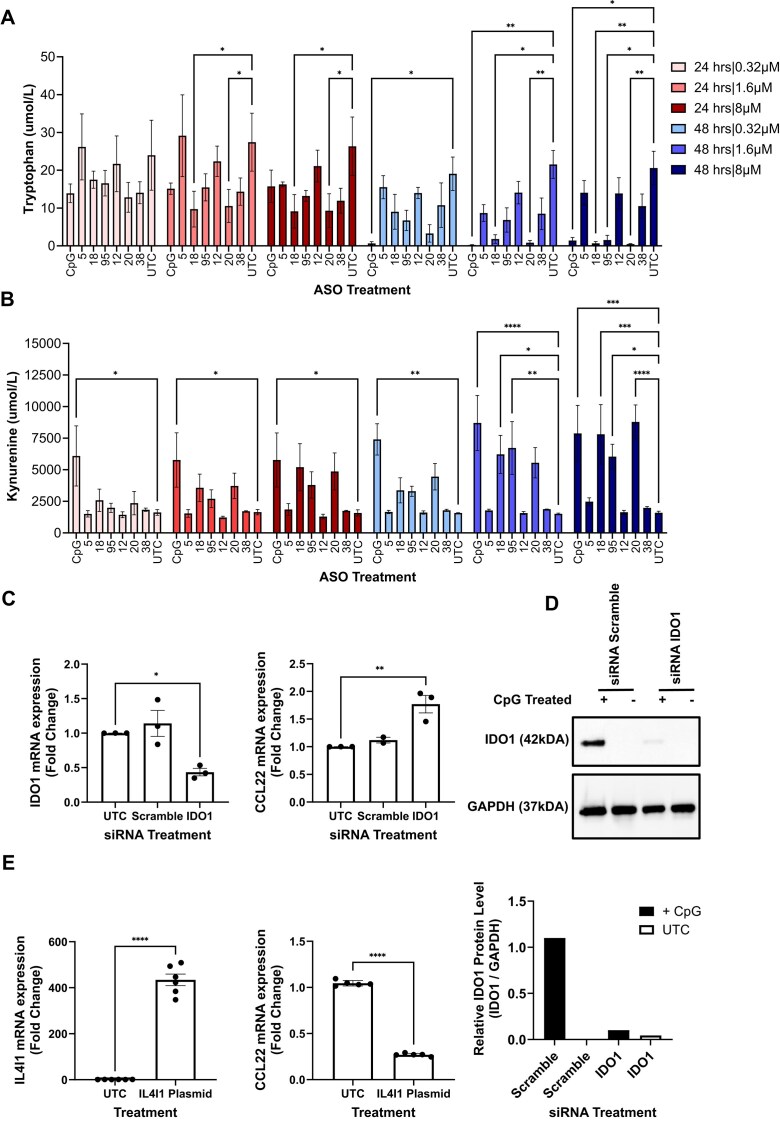
Immunogenic PS ASOs stimulate Kyn production via the tryptophan catabolism pathway, and genetic manipulation of tryptophan catabolizing enzymes alters TLR9-mediated PS ASO activation. Total extracellular (**A**) tryptophan and (**B**) kynurenine metabolite levels from THP1-TLR9 cells 24- or 48-h post-treatment with 0.32, 1.6, or 8 μM of indicated PS ASOs. (**C**) Relative CCL22 and IDO1 mRNA expression levels in IDO1 siRNA-treated THP1-TLR9 stimulated with CpG ASO. (**D**) Validation of IDO1 siRNA knockdown and representative western blot measuring IDO1 protein expression. THP1-TLR9 cells were electroporated with 1 μM of siRNA 24 h prior to treatment with 1.6 μM CpG ASO for 2 h. Protein lysates were collected 24 h following treatment. (**E**) Relative CCL22 and IL4I1 mRNA expression levels in IL4I1 overexpression plasmid-treated BJAB cells stimulated with CpG ASO. All bar graph data are expressed as mean ± S.E.M.

To confirm that the observed increase in *IDO1* mRNA expression correlates with a change in IDO1 activity, we examined the consumption of Trp and the generation of Kyn after PS ASO treatment. A dose-response study (0.32, 1.6, and 8 µM) was conducted with the PS ASOs, with measurements of Trp substrate and downstream Kyn metabolite taken at 24 and 48 h after treatment. While all PS ASOs demonstrated some decrease in Tryp, only those that concomitantly generated Kyn were considered to have induced IDO1 activity (Fig. 4A, B). A significant increase in Kyn, accompanied by a significant decrease in Trp was observed in THP1-TLR9 cells treated with rapid TLR9 agonists (CpG, ASO-20, and ASO-95), as well as ASO-18. Furthermore, we identified that peak IDO1 activity occurred 48 h after treatment for all PS ASOs, and increased with increasing PS ASO concentrations. Again, we observed that the magnitude of Tryptophan consumption and kynurenine generation correlated, albeit imperfectly, with the magnitude of TLR9 activation observed by measurement of CCL22 mRNA, complicating the SAR.

In order to understand how the genetic manipulation of IDO1 and IL4I1 affects TLR9 activation, we applied siRNA to IDO1 in THP1-TLR9 cells with and without the CpG ASO. When treated with CpG ASO, TLR9 activation was increased two-fold (Fig. 4C), and a 90% reduction in IDO1 protein was confirmed via western blot (Fig. 4D), suggesting that IDO1 plays a critical role in innate immune suppression, which may be slightly compensated for by IL4I1 in cells that express both enzymes. We chose to use an overexpression IL4I1 plasmid in the BJAB cell line to understand how TLR9 activation is altered when IL4I1 is manipulated, after considerable cell death was observed when we dosed IL4I1 siRNA in the BJAB cell line (data not shown). We achieved a 400-fold increase in IL4I1 mRNA expression, and observed a 3.3-fold decrease (or 70% reduction) in TLR9 activation when stimulated with the CpG ASO (Fig. 4E). These data provide robust evidence that IL4I1 is critical for innate immune suppression in cell lines that lack the IDO1 enzyme.

### Kynurenine pathway metabolites are upregulated, and scale with PS-ASO–driven innate immune activation

**Figure 5.**
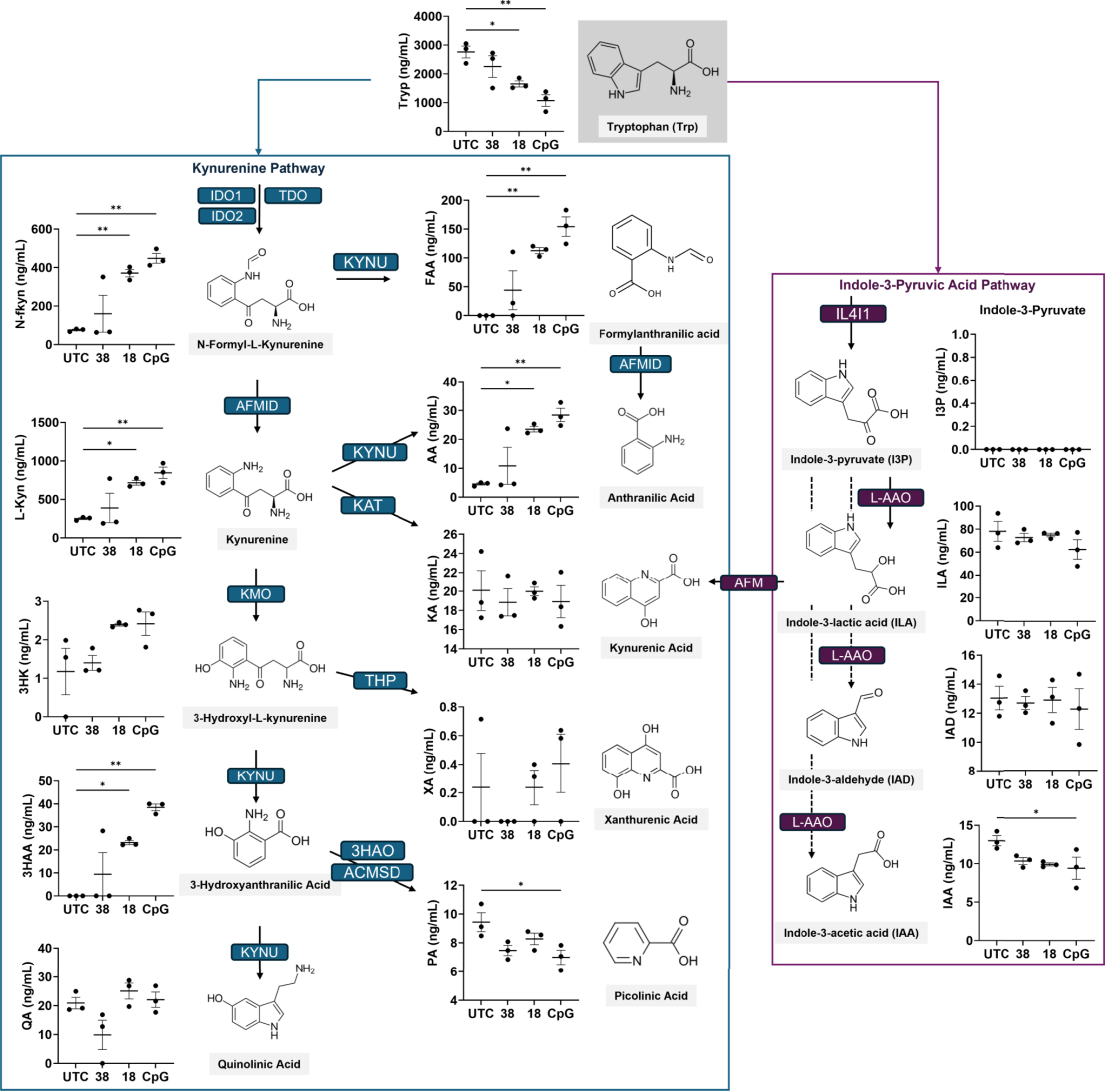
Directed metabolomics of tryptophan metabolites after PS ASO treatment in THP1-TLR9 cells. Cells were treated with 8 μM PS-ASOs for 2 h, recovered for 48 h, and the collected cellular supernatant was subjected to directed metabolomic analysis of tryptophan metabolites to quantify specific changes. Biological replicates (*n* = 3) were submitted for metabolomic analysis. Significant differences in means of metabolite quantities (ng/mL) were determined via one-way ANOVA with Dunnett post-hoc tests (statistical significance is denoted at *P* < 0.05*, *P* < 0.005**, and *P* < 0.0005***).

We next sought to explore how the profile of extracellular tryptophan and its derivatives changes when exposed to PS-ASOs of varying innate immunogenicity. THP1-TLR9 cells were treated with either the strong inflammatory CpG ASO, weaker inflammatory ASO 18, non-inflammatory ASO 38, or were untreated for 2 h. Extracellular supernatants were collected 48 h after treatment for targeted metabolomic analysis. Relative to untreated controls, tryptophan levels in the supernatant were depleted, while upstream kynurenine-pathway intermediates N-fkyn and Kyn were significantly increased in cells treated with ASO 18 and the CpG ASO, but not ASO 38, consistent with enhanced IDO1-driven tryptophan catabolism (Fig. 5). In addition, 3HK showed a trend toward higher secretion, whereas 3-HAA, FAA, and AA were all increased compared with untreated controls and ASO 38. In contrast, quinolinic acid remained unchanged, and kynurenic acid also did not vary detectably in the supernatant, likely due to the predominantly protein-bound/intracellular distribution of kynurenic acid [30]. Notably, picolinic acid, a terminal product of one KP branch, decreased, indicating less activity of this branch and diversion of metabolites into alternative KP pathway endpoints. Within the indole-3-pyruvic acid branch of tryptophan metabolism, indole-3-pyruvate itself was below the limit of detection. The AhR-active indole metabolites indole-3-lactic acid and indole-3-aldehyde were readily detected but did not change with PS-ASO treatment, whereas the related AhR agonist indole-3-acetic acid was significantly reduced, indicating its consumption. No changes in the levels of serotonin, nor its precursors, were measured. (Supplementary Fig. 4). Together, these data indicate that brief innate immune stimulation with CpG and non-CpG PS-ASOs, followed by 48 h recovery, preferentially diverts tryptophan into the kynurenine pathway while selectively depleting certain indole-derived AhR ligands, particularly indole-3-acetic acid. Interestingly, the CpG ASO typically consumed more Tryp and generated higher amounts of kyn-derived metabolites than ASO 18, which is in line with the stronger levels of innate immune activation observed in the BJAB and THP1-TLR9 cells (Fig. 3C, D). These findings suggest that the magnitude of TLR9 activation may correlate, at least in part, with the extent of downstream kynurenine-pathway engagement, offering insight into the termination processes of these PS-ASOs.

### Exogenous supplementation of kynurenine pathway metabolites suppresses PS-ASO-mediated innate immunity

**Figure 6.**
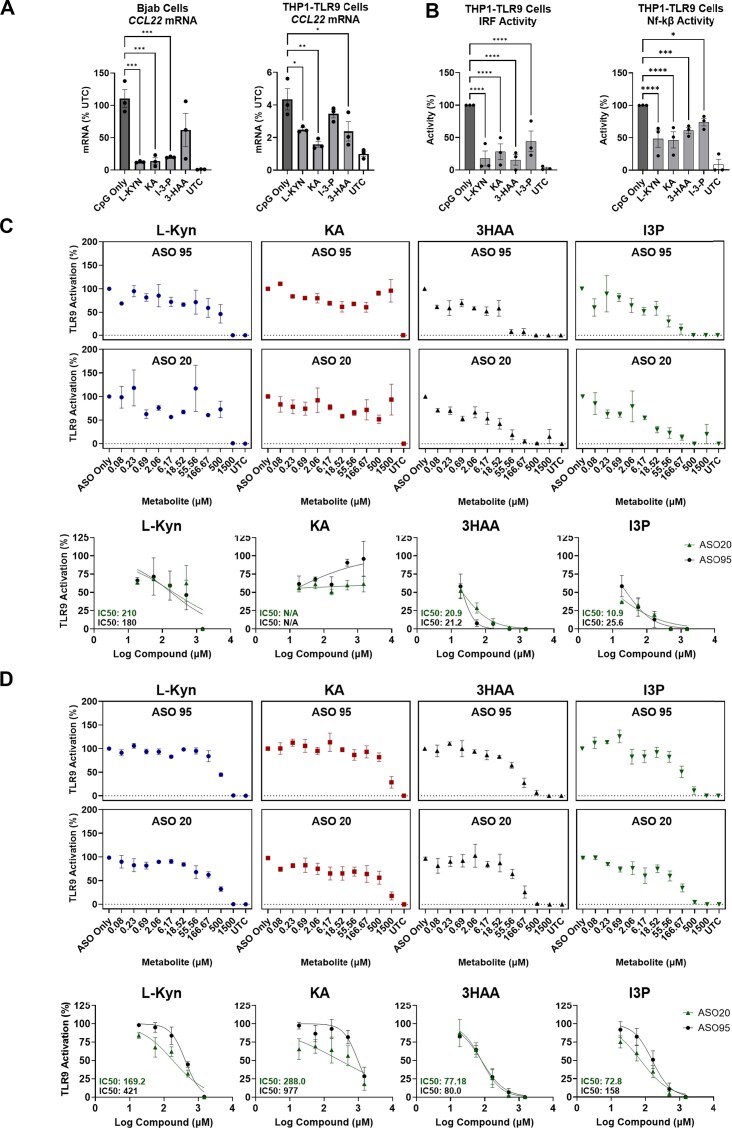
Exogenous kynurenine and indole metabolites attenuate PS-ASO-induced innate immune responses. (**A**) Relative *CCL22* mRNA expression levels in BJAB and THP1-TLR9 cells and (**B**) Relative IRF reporter activity and NF-kB reporter activity in THP1-TLR9 cells following treatment with 100 µM of the indicated metabolite and 0.8 µM CpG ASO. Relative TLR9 activation of (**C**) THP1-TLR9 cells and (**D**) BJAB cells pretreated with various doses (1.5 mM–0.08 µM) of kynurenine, kynurenic acid, 3-hydroxy anthranilic acid, and indole-3-pyruvate for 2 h, and subsequently treated with 1.6 µM of ASO-20 or ASO-95 for 24 h. TLR9 activation was measured as *CCL22* mRNA expression. Metabolite IC50s derived from the top five doses are shown for each ASO, calculated using linear regression with normalized response and variable slope. All experiments were reproduced in at least three independent biological experiments, with multiple replicates per experiment.

It has previously been demonstrated that administration of key kynurenine pathway metabolites, including Indole-3-pyruvic acid, Kynurenine, and Kynurenic acid, modulates the immune response to maintain immune tolerance by activating the AhR receptor [31]. We therefore asked whether exogenous AhR ligands could be applied *in vitro* to dampen PS-ASO-induced innate immune activation in monocytic and lymphoid cell lines. Concomitant treatment of BJAB and THP1-TLR9 cells with 100 µM Kyn, KYNA, I3P, and 3-HAA reduced *CCL22* mRNA induction following PS-ASO stimulation (Fig. 6A). In THP1-TLR9 cells, this reduction in *CCL22* mRNA was consistent with decreased IRF and NF-κB reporter activity in the presence of these AhR ligands (Fig. 6B).

To determine whether this innate immune suppression was specific to a CpG-containing PS-ASO or generalizable to other TLR9-activating chemistries, we extended these studies to ASO 20 and ASO 95, which lack CpG motifs but still bind and activate TLR9. Each AhR ligand was tested in a dose–response series from 1.5 mM down to 0.08 µM in THP1-TLR9 cells (Fig. 6C) and BJAB cells (Fig. 6D), with IC₅₀ values calculated from the top five concentrations. In THP1-TLR9 cells, I3P was the most potent suppressor, with IC₅₀ values of 10.9 µM for ASO 20 and 25.6 µM for ASO 95, followed by 3-HAA with IC₅₀ values of 20.9 µM and 21.2 µM for ASO 20 and ASO 95, respectively. L-kynurenine was less potent, with IC₅₀ values of 210 µM and 180 µM for ASO 20 and ASO 95, respectively. Kynurenic acid did not show appreciable suppressive effects against ASO 20 or ASO 95 in THP1-TLR9 cells at the concentrations tested, which may reflect high serum binding and variable uptake kinetics of KA in this system.

In BJAB cells, IC₅₀ values were generally higher but followed the same rank order of potency. 3-HAA and I3P again exhibited the lowest IC₅₀ values: 3-HAA had IC₅₀ values of 77.18 µM (ASO 20) and 80.0 µM (ASO 95), whereas I3P had IC₅₀ values of 72.8 µM (ASO 20) and 158 µM (ASO 95). L-kynurenine was less potent in BJAB cells, with IC₅₀ values of 169.2 µM and 421 µM for ASO 20 and ASO 95, respectively. Interestingly, in contrast to the THP1-TLR9 model, kynurenic acid did exert immunosuppressive effects in BJAB cells, albeit with relatively high IC₅₀ values of 288.0 µM for ASO 20 and 977 µM for ASO 95. Overall, these data provide evidence that KP metabolites robustly dampen PS ASO innate immune responses, thereby increasing cellular tolerance to immunogenic PS ASOs.

## Discussion

Our study provides a mechanistic framework for understanding how PS-ASO-induced innate immune activation interfaces with the tryptophan oxidation pathways governed by IDO1 and IL4I1. By comparing PS-ASOs with distinct innate immune activation profiles across two complementary human immune cell systems, we show that TLR9-driven responses consistently engage these enzymes and remodel the downstream metabolite landscape. These findings extend earlier observations that IDO1 and IL4I1 are broadly inducible by inflammatory signals, and position these enzymes as modulators of the magnitude of PS-ASO-induced innate signaling rather than simple markers of inflammation.

We identify distinct classes of PS ASOs that differ in cytokine kinetics, providing a more nuanced understanding of their immune-modulating properties. Notably, we show that both fast and slow TLR9-dependent agonists identified by Pollak et al. (2022) [5, 6] activate the compensatory kynurenine pathway (KP) through the upregulation of key tryptophan-metabolizing enzymes, IDO1 and IL4I1. This extends previous reports of TLR9-mediated IDO1 induction and subsequent innate immune suppression [25, 32, 33, 34], by demonstrating that TLR9 activation by PS ASOs in both lymphoid and myeloid cells leads to rapid and quantifiable accumulation of not only IDO1, but also IL4I1 in lymphoid cells that lack IDO1. In addition, we characterize the kinetics and scale of enzyme induction. This was supported by targeted metabolite quantification through both ELISA and metabolomics, where the production of kynurenine and its derivatives, along with tryptophan depletion, strengthened the link between the KP and PS-ASO immune tolerance. Moreover, manipulation of IDO1, responsible for 90% of tryptophan conversion to KYNA, led to an enhanced immune response, underscoring its critical role in immune modulation; in addition, overexpression of IL4I1 in lymphoid cells that lack IDO1 led to immune suppression. Finally, we demonstrate that pre-treatment with various kynurenine and indole-derived metabolites can increase immune tolerance for various PS ASOs by suppressing the magnitude of the induced innate immune response. Collectively, our study builds upon compelling evidence that the KP serves as a key terminator of PS ASO-induced innate immune activation in specific cell types, and that metabolites of this pathway may be used to enhance immune tolerance to innate immune activating PS ASOs. Considering IDO1 induction is a well-established downstream consequence of multiple TLR pathways, the tryptophan-catabolic feedback loop described here may represent a broader innate immune attenuation mechanism engaged whenever PS ASOs activate pattern-recognition receptors. These findings advance our understanding of immune regulation by PS ASOs and may inform strategies to optimize their therapeutic safety and efficacy.

Variations in innate immune activation observed between THP1-TLR9 and BJAB cell lines likely arise due to differences in abundance, expression patterns, and subunit organization of TLR9. Given that the THP1-TLR9 cell lines are transgenic for TLR9 receptors, a lack of natural receptor configurations, densities, and lack of native regulatory elements may lead to underrepresentation of TLR9 agonist PS ASO-mediated immunogenic responses. Furthermore, differences in cell TLR9 signatures would ostensibly impact TLR9-mediated signaling cascades. TLR9 activation typically generates a “cytokine storm” comprised of various pro-inflammatory cytokines and chemokines, resulting in a specific immune response signature [35, 36]. Distinct immune signatures after PS ASO stimulation in the BJAB and THP1-TLR9 cell lines are also unsurprising, given the innate differences in the lineage of each immune cell type; THP1 cells are monocytic and readily differentiate to dendritic cells to generate chemokines upon TLR9 activation [37, 38, 39], whereas BJAB cells are an analog of regulatory B cells that primarily secrete immunosuppressive cytokines [28, 40, 41]. When our data is considered in the context of published results [5, 6, 26], it is tempting to conclude that PS ASO-induced innate immune activation is strictly due to interactions with TLR9 receptors in late endosomes (LE). However, we demonstrated significant differences in response to PS ASOs in the two cell lines studied. A recent report demonstrated that synthetic 2′-O-methyl modified short oligonucleotides can interact with TLR7 and TLR8 binding sites [42], which may explain some differential response observed between oligos and cell lines; however, previous knockout studies have demonstrated that TLR9 is responsible for innate immune activation within the Bjab cell system [20]. Future *in vivo* studies will help further elucidate the breadth of pathways that modulate PS-ASO-induced signaling. As TLR9 receptors are located in the LE, and these organelles accumulate high concentrations of PS ASOs [43], it’s worth considering whether cytoplasmic sensors are activated if PS ASOs accumulation in the LE is either blocked or oversaturated.

We hypothesized that the strength and kinetics of innate immune suppression processes correlate with the strength and kinetics of PS ASO-induced innate activation. Indeed, we show that there are imperfect correlations between the intensity and timing of immune activation by PS ASOs, which raises an interesting question. For instance, can some of the complex SAR of PS ASO TLR9 activation be ascribed to a more robust suppression signal for partial agonists? Thus, any outliers may be of particular interest. Nonetheless, the data presented here clearly demonstrate a solid connection between TLR9 activation and the upregulation of IDO1 and IL4I1 in response to both fast and slow PS ASO-TLR9 agonists, as well as partial agonists, across two cell systems. Moreover, the kinetics of this relationship change with ASO chemistry and sequence. Alterations in the kinetics are likely due to subtle differences at the TLR9 receptor, as PS ASO-TLR9 receptor engagement is chemistry and sequence-dependent [6, 44]. In addition, the CpG ASO, which exhibits stronger TLR9 activation, was also found to drive a more pronounced tryptophan depletion and KP metabolite accumulation than ASO 18, suggesting that sequence and motif-dependent TLR9 agonism is coupled to the extent of downstream immune-metabolic engagement.

The KP and its metabolites have gained interest as potential immune regulatory targets in the clinic. Specifically, KP modulators delivered in combination with other therapeutics may improve patient outcomes by limiting side effects in a variety of systemic inflammatory diseases, such as cancers, and neurodegenerative conditions [45–49, 50]. Given the identification of certain immunogenic PS ASOs [24], we were particularly interested in the response of the KP to treatment with PS ASOs, and the identification of key metabolites that pose as druggable candidates for co-therapy in mitigating systemic inflammation. Our results confirm that exogenous KP metabolites, but also the Indole pathway metabolites, are likely targets for increasing the tolerability of immunogenic PS ASOs. This conclusion is supported by increased production of KP metabolites in THP1-TLR9 cells treated with TLR9 agonists, which appear to scale with increasing innate immunogenicity of the PS-ASO applied, for both the ELISA and metabolomics data. It should be noted, however, that slight discrepancies between recruitment of the KP and subsequent production of immune-quenching Kyn appeared independent of the strength of TLR9 activation, as even the weak TLR9 agonist, ASO-18, produced Kyn levels analogous to levels of strong agonists. We hypothesize that there may be a low threshold for KP activation and rapid recruitment of immunomodulatory metabolites, particularly given the highly conserved nature of this pathway [51]. Further to this, silencing IDO1 increased the innate immune response detectable with CpG-treated THP1-TLR9 cells, and conversely, overexpressing IL4I1 decreased the innate immune response detectable with CpG-treated BJAB cells. This corroborates current literature indicating a critical role for IDO1 as an immune suppressant, via upstream modulation of Kyn generation, which can no longer engage the AhR [52], and provides evidence that in the absence of IDO1, IL4I1 can act as an immune suppressant[17]. Finally, we also observed robust, dose-dependent suppression of TLR9 activation in both cell systems using various exogenous KP and Indole metabolites, to both CpG and non-CpG ASOs. We conclude that the KP and indole oxidative metabolites likely activate the immunosuppressant functions of AhR via the production of Kyn, leading to dampening of the PS ASO-mediated innate immune response.

Importantly, our findings offer potential translational implications. Immunogenic PS ASOs are known to trigger acute cytokine elevations in humans, and strategies that blunt these responses could improve tolerability and broaden therapeutic windows. The observation that kynurenine- and indole-derived metabolites reduce innate immune activation suggests opportunities to co-formulate PS ASOs with metabolic modulators or employ transient metabolic supplementation to dampen unwanted proinflammatory responses. While further *in vivo* studies are required, this work provides a foundation for exploring such strategies.

Taken together, we identified and characterized immune signatures of PS ASO-TLR9 agonists in two cell lines, highlighting the critical role of the key tryptophan-catabolizing enzymes, IDO1 and IL4I1, in generating KP metabolites to quench the immune response. This work opens new avenues for exploring KP modulation to mitigate the risk of PS ASO-induced innate immune activation. In the clinic, it may be possible to include KP metabolites as excipients in PS ASO formulations or as a co-therapy, although further work is needed. In addition, although validated as a surrogate readout of PS ASO innate immunogenicity, the BJAB/CCL22 assay has some limitations and does not fully capture the complex molecular milieu of immune responses due to PS ASOs. Future work utilizing time course, untargeted RNAseq, and proteomics would be valuable extensions to broaden pathway discovery beyond the Tryptophan-IDO1/IL4I1 axis explored in this study. Finally, whilst siRNA and plasmid overexpression studies provide valuable mechanistic insights into the effects of various genes, enzymes, and proteins, future work should explore the use of IDO1/IL4I1 single and double knockout models for further exploration, if found to be viable. Nevertheless, this study provides compelling evidence that the KP and indole pathway play a mechanistic role in PS ASO-mediated immune regulation, paving the way for future research. These insights advance our understanding of how cells regulate innate immune responses to PS ASOs and highlight actionable avenues for enhancing the tolerability of next-generation antisense therapeutics.

## Supplementary Material

gkag311_Supplemental_File

## Data Availability

The data underlying this article are available in the article and in its online supplementary material.

## References

[1] Crooke ST, Wang S, Vickers TA et al. Cellular uptake and trafficking of antisense oligonucleotides. Nat Biotechnol. 2017;35:230–7. 10.1038/nbt.3779. 28244996

[2] Crooke ST, Liang XH, Baker BF et al. Antisense technology: a review. J Biol Chem. 2021;296:100416. 10.1016/j.jbc.2021.100416. 33600796 PMC8005817

[3] Crooke ST, Baker BF, Crooke RM et al. Antisense technology: an overview and prospectus. Nat Rev Drug Discov. 2021;20:427–53. 10.1038/s41573-021-00162-z. 33762737

[4] Pollak AJ, Zhao L, Crooke ST. Systematic analysis of chemical modifications of phosphorothioate antisense oligonucleotides that modulate their innate immune response. Nucleic Acid Ther. 2023;33:95–107. 10.1089/nat.2022.0067. 36749166

[5] Pollak AJ, Zhao L, Vickers TA et al. Insights into innate immune activation via PS-ASO-protein-TLR9 interactions. Nucleic Acids Res. 2022;50:8107–26. 10.1093/nar/gkac618. 35848907 PMC9371907

[6] Pollak AJ, Zhao L, Crooke ST. Characterization of cooperative PS-oligo activation of human TLR9. Mol Ther - Nucleic Acids. 2023;33:832–44. 10.1016/j.omtn.2023.08.011. 37675184 PMC10477407

[7] Pereira M, Gazzinelli RT. Regulation of innate immune signaling by IRAK proteins. Front. Immunol. 2023;14:1133354. 10.3389/fimmu.2023.1133354. 36865541 PMC9972678

[8] Kawai T, Ikegawa M, Ori D et al. Decoding toll-like receptors: recent insights and perspectives in innate immunity. Immunity. 2024;57:649–73. 10.1016/j.immuni.2024.03.004. 38599164

[9] Mezrich J.D., Fechner J.H., Zhang X et al. An interaction between kynurenine and the aryl hydrocarbon receptor can generate regulatory T cells. J Immunol. 2010;185:3190–8. 10.4049/jimmunol.0903670. 20720200 PMC2952546

[10] Yeung AW, Terentis AC, King NJ et al. Role of indoleamine 2,3-dioxygenase in health and disease. Clin Sci (Lond). 2015;129:601–72. 10.1042/CS20140392. 26186743

[11] Orhan F., Bhat M., Sandberg K et al. Tryptophan metabolism along the kynurenine pathway downstream of toll-like receptor stimulation in peripheral monocytes. Scand J Immunol. 2016;84:262–71. 10.1111/sji.12479. 27607184

[12] Badawy AA, Guillemin G. The plasma [Kynurenine]/[Tryptophan] ratio and indoleamine 2,3-dioxygenase: time for appraisal. Int J Tryptophan Res. 2019;12:1178646919868978. 10.1177/1178646919868978. 31488951 PMC6710706

[13] Kor A, Erten Ş., Yurt EF et al. Clinical significance of plasma tryptophan, kynurenine, and kynurenine/tryptophan ratio in rheumatoid arthritis patients. The Egyptian Rheumatologist. 2022;44:367–71. 10.1016/j.ejr.2022.07.005.

[14] Sorgdrager FJH, Naude PJW, Kema IP et al. Tryptophan metabolism in inflammaging: from biomarker to therapeutic target. Front. Immunol. 2019;10:2565. 10.3389/fimmu.2019.02565. 31736978 PMC6833926

[15] Sun J, Shi J, Li J et al. The effect of immunosuppressive adjuvant kynurenine on type 1 diabetes vaccine. Front. Immunol. 2021;12:681328. 10.3389/fimmu.2021.681328. 34305913 PMC8293994

[16] Zuo M, Fang J, Huang P et al. IL4I1-catalyzed tryptophan metabolites mediate the anti-inflammatory function of cytokine-primed human muscle stem cells. Cell Death Discov. 2023;9:269. 10.1038/s41420-023-01568-x. 37507432 PMC10382538

[17] Zeitler L, Murray PJ. IL4i1 and IDO1: oxidases that control a tryptophan metabolic nexus in cancer. J Biol Chem. 2023;299:104827. 10.1016/j.jbc.2023.104827. 37196768 PMC10318530

[18] Pollak AJ, Cauntay P, Machemer T et al. Mechanism driven early stage identification and avoidance of antisense oligonucleotides causing TRL9 mediated inflammatory responses in Bjab cells. bioRxiv, 10.1101/2021.12.12.472280, 14 December 2021, preprint: not peer reviewed.

[19] Anderson BA, Freestone GC, Low A et al. Towards next generation antisense oligonucleotides: mesylphosphoramidate modification improves therapeutic index and duration of effect of gapmer antisense oligonucleotides. Nucleic Acids Res. 2021;49:9026–41. 10.1093/nar/gkab718. 34417625 PMC8450106

[20] Pollak AJ, Cauntay P, Machemer T et al. Inflammatory non-CpG antisense oligonucleotides are signaling through TLR9 in Human burkitt lymphoma B bjab cells. Nucleic Acid Ther. 2022;32:473–85. 10.1089/nat.2022.0034. 36355073

[21] Partridge W, Burel SA, Ferng A et al. Correlations between preclinical BJAB assay ranking of antisense drugs and clinical trial adverse events. Clinical Translational Sci. 2023;16:575–80. 10.1111/cts.13476. PMC10087069 36631935

[22] Henry SP, Arfvidsson C, Arrington J et al. Assessment of the immunogenicity potential for oligonucleotide-based drugs. Nucleic Acid Ther. 2022;32:369–77. 10.1089/nat.2021.0112. 36178478

[23] Bauer S, Kirschning C.J, Hacker H et al. Human TLR9 confers responsiveness to bacterial DNA via species-specific CpG motif recognition. Proc. Natl. Acad. Sci. U.S.A. 2001;98:9237–42. 10.1073/pnas.161293498. 11470918 PMC55404

[24] Burel SA, Machemer T, Baker BF et al. Early-stage identification and avoidance of antisense oligonucleotides causing species-specific inflammatory responses in Human volunteer peripheral blood mononuclear cells. Nucleic Acid Ther. 2022;32:457–72. 10.1089/nat.2022.0033. 35976085

[25] Mellor AL, Baban B, Chandler PR et al. Cutting edge: cpG oligonucleotides induce splenic CD19+ dendritic cells to acquire potent indoleamine 2,3-dioxygenase-dependent T cell regulatory functions via IFN type 1 signaling. J Immunol. 2005;175:5601–5. 10.4049/jimmunol.175.9.5601. 16237046

[26] Liang XH, Nichols JG, De Hoyos CL et al. Golgi-58K can re-localize to late endosomes upon cellular uptake of PS-ASOs and facilitates endosomal release of ASOs. Nucleic Acids Res. 2021;49:8277–93. 10.1093/nar/gkab599. 34244781 PMC8373082

[27] Liang XH, Sun H, Nichols JG et al. COPII vesicles can affect the activity of antisense oligonucleotides by facilitating the release of oligonucleotides from endocytic pathways. Nucleic Acids Res. 2018;46:10225–45. 10.1093/nar/gky841. 30239896 PMC6212795

[28] Chang J, Kunkel SL, Chang CH. Negative regulation of MyD88-dependent signaling by IL-10 in dendritic cells. Proc. Natl. Acad. Sci. U.S.A. 2009;106:18327–32. 10.1073/pnas.0905815106. 19815506 PMC2775313

[29] Chanput W, Peters V, Wichers H. THP-1 and U937 cells. In: Verhoeckx K, Cotter P, López-Expósito I, et al. (eds.), The Impact of Food Bioactives on Health: In Vitro and Ex Vivo Models. Cham (CH): Springer, 2015. 29787039

[30] Alves LF, Moore J.B, Kell DB. The biology and biochemistry of Kynurenic acid, a potential nutraceutical with multiple biological effects. Int J Mol Sci. 2024;25:9082, 10.3390/ijms25169082 . 39201768 PMC11354673

[31] Aoki R, Aoki-Yoshida A, Suzuki C et al. Indole-3-Pyruvic acid, an aryl hydrocarbon receptor activator, suppresses experimental colitis in mice. J Immunol. 2018;201:3683–93. 10.4049/jimmunol.1701734. 30429284

[32] Munn DH, Sharma M.D, Lee JR et al. Potential regulatory function of human dendritic cells expressing indoleamine 2,3-dioxygenase. Science. 2002;297:1867–70. 10.1126/science.1073514. 12228717

[33] Wingender G, Garbi N, Schumak B et al. Systemic application of CpG-rich DNA suppresses adaptive T cell immunity via induction of IDO. Eur J Immunol. 2006;36:12–20. 10.1002/eji.200535602. 16323249

[34] Fallarino F, Puccetti P. Toll-like receptor 9-mediated induction of the immunosuppressive pathway of tryptophan catabolism. Eur J Immunol. 2006;36:8–11. 10.1002/eji.200535667. 16358364

[35] Ghosh TK, Mickelson DJ, Fink J et al. Toll-like receptor (TLR) 2-9 agonists-induced cytokines and chemokines: I. Comparison with T cell receptor-induced responses. Cell Immunol. 2006;243:48–57. 10.1016/j.cellimm.2006.12.002. 17250816

[36] Saber MM, Monir N, Awad AS et al. TLR9: a friend or a foe. Life Sci. 2022;307:120874. 10.1016/j.lfs.2022.120874. 35963302

[37] Decalf J, Fernandes S, Longman R et al. Plasmacytoid dendritic cells initiate a complex chemokine and cytokine network and are a viable drug target in chronic HCV patients. J Exp Med. 2007;204:2423–37. 10.1084/jem.20070814. 17893202 PMC2118448

[38] Hatscher L, Amon L, Heger L et al. Inflammasomes in dendritic cells: friend or foe?. Immunol Lett. 2021;234:16–32. 10.1016/j.imlet.2021.04.002. 33848562

[39] Holken JM, Teusch N. The monocytic cell line THP-1 as a validated and robust surrogate model for human dendritic cells. Int J Mol Sci. 2023;2:1452. 10.3390/ijms24021452 PMC9866978 36674966

[40] Upasani V, Rodenhuis-Zybert I, Cantaert T. Antibody-independent functions of B cells during viral infections. PLoS Pathog. 2021;17:e1009708. 10.1371/journal.ppat.1009708. 34293057 PMC8297758

[41] Rastogi I, Jeon D, Moseman JE et al. Role of B cells as antigen presenting cells. Front Immunol. 2022;13:954936. 10.3389/fimmu.2022.954936. 36159874 PMC9493130

[42] Alharbi AS, Sapkota S, Zhang Z et al. 2'-O-methyl-guanosine RNA fragments antagonize TLR7 and TLR8 to limit autoimmunity. Nat Immunol. 2026;27:762–75. 10.1038/s41590-026-02429-2. 41667621 PMC13043311

[43] Wang S, Sun H, Tanowitz M et al. Intra-endosomal trafficking mediated by lysobisphosphatidic acid contributes to intracellular release of phosphorothioate-modified antisense oligonucleotides. Nucleic Acids Res. 2017;45:5309–22. 10.1093/nar/gkx231. 28379543 PMC5605259

[44] Drygin D, Koo S, Perera R et al. Induction of toll-like receptors and NALP/PAN/PYPAF family members by modified oligonucleotides in lung epithelial carcinoma cells. Oligonucleotides. 2005;15:105–18. 10.1089/oli.2005.15.105. 15989425

[45] Swardfager W, Herrmann N, Dowlati Y et al. Indoleamine 2,3-dioxygenase activation and depressive symptoms in patients with coronary artery disease. Psychoneuroendocrinology. 2009;34:1560–6. 10.1016/j.psyneuen.2009.05.019. 19540675

[46] Nakajima K, Yamashita T, Kita T et al. Orally administered eicosapentaenoic acid induces rapid regression of atherosclerosis via modulating the phenotype of dendritic cells in LDL receptor-deficient mice. ATVB. 2011;31:1963–72. 10.1161/ATVBAHA.111.229443. 21817104

[47] Opitz CA, Litzenburger UM, Opitz U et al. The indoleamine-2,3-dioxygenase (IDO) inhibitor 1-methyl-D-tryptophan upregulates IDO1 in human cancer cells. PLoS One. 2011;6:e19823. 10.1371/journal.pone.0019823. 21625531 PMC3098827

[48] Sundaram G, Lim CK, Brew BJ et al. Kynurenine pathway modulation reverses the experimental autoimmune encephalomyelitis mouse disease progression. J Neuroinflammation. 2020;17:176. 10.1186/s12974-020-01844-y. 32505212 PMC7276083

[49] Lashgari NA, Roudsari NM, Shayan M et al. IDO/Kynurenine; novel insight for treatment of inflammatory diseases. Cytokine. 2023;166:156206. 10.1016/j.cyto.2023.156206. 37120946

[50] Chen CM, Huang CY, Lai CH et al. Neuroprotection effects of kynurenic acid-loaded micelles for the Parkinson’s disease models. J Liposome Res. 2024;34:593–604. 10.1080/08982104.2024.2346986. 38779944

[51] Savitz J . The kynurenine pathway: a finger in every pie. Mol Psychiatry. 2020;25:131–47. 10.1038/s41380-019-0414-4. 30980044 PMC6790159

[52] Cheong JE, Sun L. Targeting the IDO1/TDO2-KYN-AhR pathway for cancer immunotherapy - challenges and opportunities. Trends Pharmacol Sci. 2018;39:307–25. 10.1016/j.tips.2017.11.007. 29254698

