# Supplementary material for: Phosphorothioate antisense oligonucleotide induced innate immune activation is attenuated by tryptophan oxidation products
Source: Nucleic Acids Res. 2026 Apr 13;54(7):gkag311. doi: 10.1093/nar/gkag311 (PMC13076217; doi:10.1093/nar/gkag311)
Supplement: gkag311_Supplemental_File [file gkag311_supplemental_file.pdf]

SUPPLEMENTARY DATA

SUPPLEMENTARY TABLES

Supplementary Table 1. Primer probe sets used in this study

Assay ID	Gene Target	Supplier, Cat#
Hs00541746_m1	IL4I1	Thermo Fisher Scientific, 4351370
Hs00984148_m1	IDO1	Thermo Fisher Scientific, 4351370

Supplementary Table 2. siRNA used in this study

siRNA	Supplier	Catalog number
IDO1 siRNA (h)	Santa Cruze Biotechnology	sc-45939
siRNA-A	Santa Cruze Biotechnology	sc-37007

Supplementary Table 3. Lumit and ELISA assays used in this study

Assay ID	Supplier	Catalog Number
Lumit(TM) Human IL-1beta Immunoassay	Promega	W6010
Lumit(TM) TNF-alpha (Human) Immunoassay	Promega	W6050
Lumit(TM) IL-6 (Human) Immunoassay	Promega	W6030
Lumit(TM) IL-10 (Human) Immunoassay	Promega	W6070
Kynurenine / Tryptophan ratio ELISA pack I High Sensitivity	Immusmol	ISE-2227

Supplementary Table 4. Antibodies used within this study

Antibody	Supplier	Catalog Number

IDO (D5J4E™) Rabbit mAb	Cell Signaling Technology	86630S
IL4I1 Human Antibody	R&D Systems	MAB5684
GAPDH Antibody (G-9)	Santa Cruz Biotechnology	sc-365062
Goat Anti-Rabbit IgG (H + L)-HRP Conjugate	Bio-Rad	1706515
Goat Anti-Mouse IgG (H + L)-HRP Conjugate	Bio-Rad	1706516

Additional Primer-probe sets:

CCL22 Fwd: CGCGTGGTGAAACACTTCTA

CCL22 Rvs: GATCGGCACAGATCTCCTTATC

CCL22 Probe: /56-FAM/TGGCGTGGT/ZEN/GTTGCTAACCTTCA/3IABkFQ/

Supplementary Table 5. Cell line characteristics

Cell Line	Characteristics			Expression*			
	Hematopoietic Lineage	Disease Class	Subtype	TLR9	CCL22	IDO1	IL4I1
BJAB	Lymphoid	Mature B-Cell Neoplasms	Burkitt Lymphoma	4.9	1.3	0	0.48
KARPAS1718	Lymphoid	Mature B-Cell Neoplasms	Splenic Marginal Zone Lymphoma	3.7	2.43	0	0.21
THP1	Myeloid	Acute Myeloid Leukemia	Acute Myeloid Leukemia	1.3	2.0	0.04	1.00
MONOMAC1	Myeloid	Acute Myeloid Leukemia	Acute Monoblastic/ Monocytic Leukemia	2.3	0.38	0.1	4.2

*Expression values are log_2(TPM + 1) RNA-seq values from Expression Public 25Q3; Data source: DepMap, Broad Institute. DepMap Public 25Q3 (2025). Available at: https://depmap.org/portal/[1]

SUPPLEMENTARY FIGURES

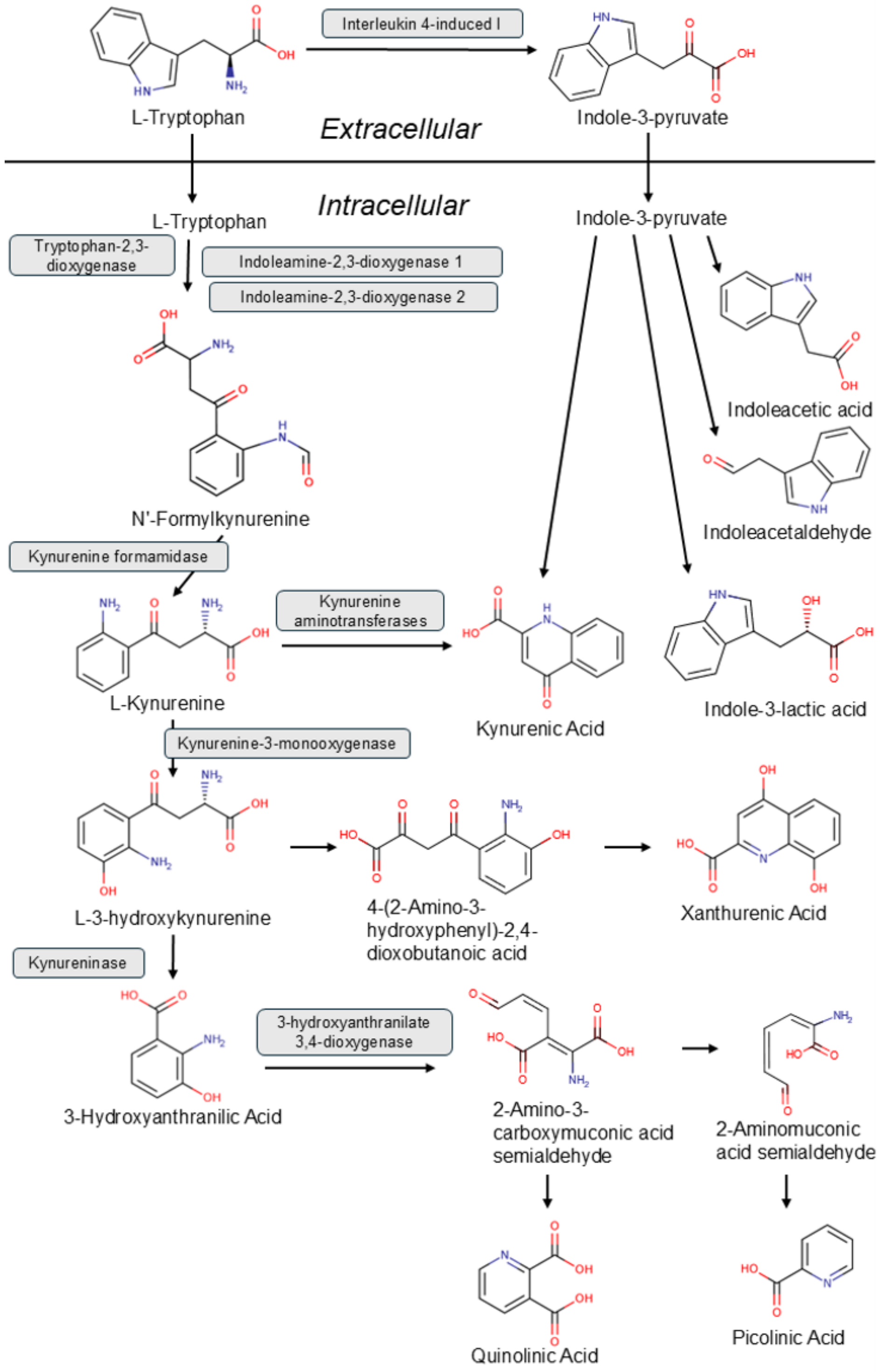

Supplementary Figure 1. Summary of Trp oxidation via IDO1 and IL4I1. Intracellular IDO1 oxidizes Trp to generate N-formyl-kynurenine, which gives rise to kyn. Kyn catabolizes into multiple Trp metabolites, including Kynurenic Acid, Xanthurenic Acid, Quinolinic Acid, and Picolinic Acid. IL4I1 is

a secreted enzyme that can catalyze Trp into indole-3-pyruvate. Indole-3-pyruvate circulates in the extracellular milieu, giving rise to metabolites such as kynurenic acid, indoleacetic acid, indoleacetaldehyde, and indole-3-lactic acid. 2-D Molecular structures were retrieved from HMDB online. L-Trp (HMDB0000929); N'-Formylkynurenine (HMDB01200); L-Kynurenine (HMDB0000684); L-3-Hydroxykynurenine (HMDB0011631); 3-Hydroxyanthranilic acid (HMDB0001476); 2-Amino-3-carboxymuconic acid semialdehyde (HMDB0001330); Quinolinic acid (HMDB0000232); Picolinic acid (HMDB0002243); 2-Aminomuconic acid semialdehyde (HMDB0001280); 4-(2-Amino-3-hydroxyphenyl)-2,4-dioxobutanoic acid (HMDB0004083); Xanthurenic acid (HMDB0000881); Kynurenic acid (HMDB0000715); Indolepyruvate (HMDB0060484); Indoleacetic acid (HMDB0000197); Indoleacetaldehyde (HMDB0001190).

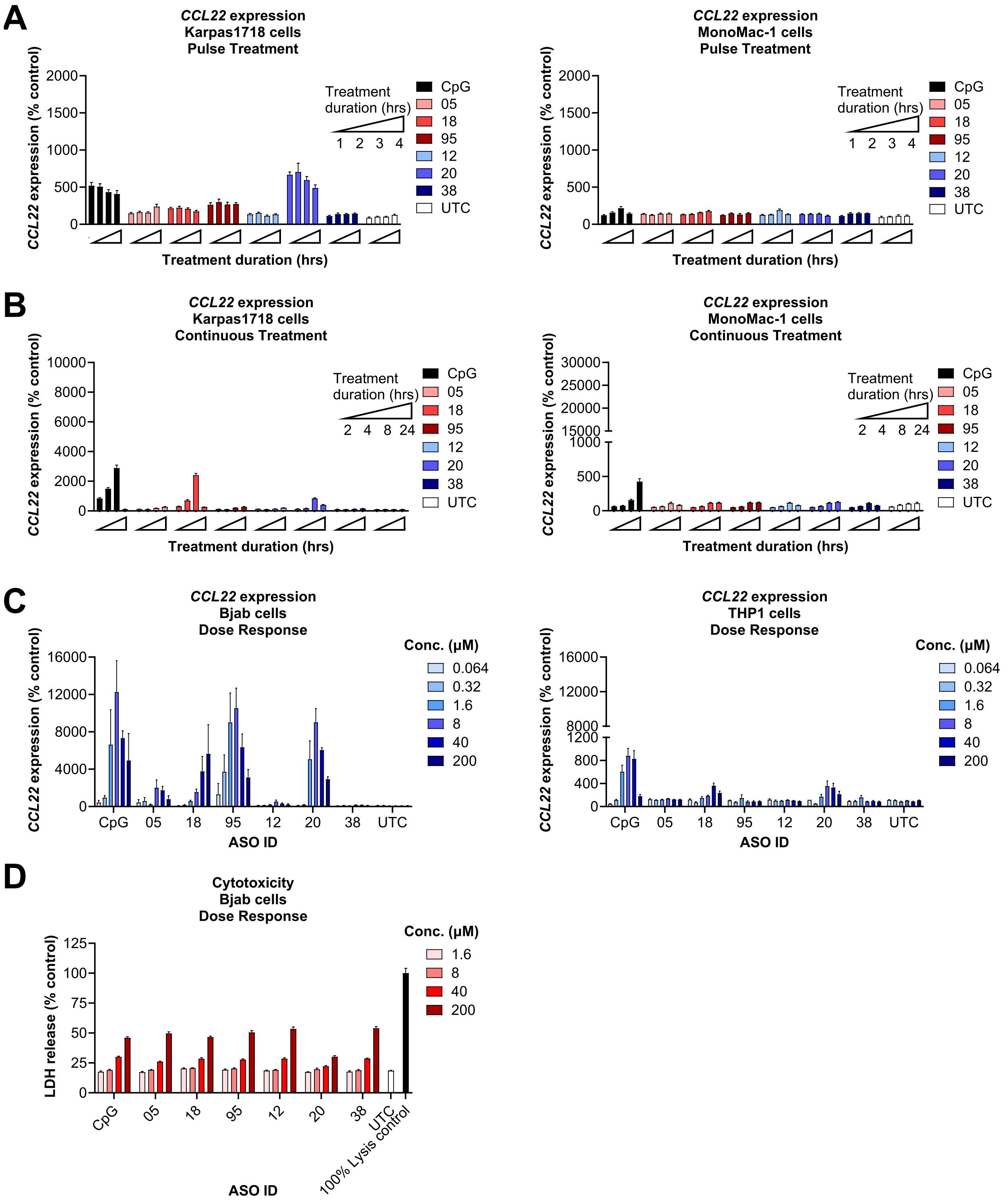

Supplementary Figure 2. Karpas-1718 and MonoMac-1 are unresponsive to a broad range of PS-ASOs and high PS-ASO doses lead to cell death. (A) Pulse treatment in Karpas1718 cell line (left) and MonoMac-1 cell line (right). Relative qRT-PCR levels of CCL22 mRNA following a 1, 2, 3, or

4 hour incubation with 1.6 μM of indicated PS-ASOs in serum-free RPMI media. RNA lysates were collected 24 hours following treatment. (B) Continuous treatment in Karpas1718 cell line(left) and MonoMac-1 cell line (right). Relative qRT-PCR levels of CCL22 following a 2, 4, 8, or 24 hour incubation with 1.6 μM of indicated PS-ASOs in serum-free RPMI media. RNA lysates were collected immediately following treatment. (C) Dose response in Bjab cell line (left) or THP1-TLR9 cell line (right). Cells were treated for 2 hours with varying concentrations (0.064, 0.32, 1.6, 8, 40, or 200 μM) of PS-ASOs. RNA lysates were collected 24 hours after treatment. (D) Cell death after PS-ASO dose response in Bjab cell line. Cells were treated with indicated concentrations of PS-ASOs for 2 hours. Cell culture supernatants were collected 24 hours after treatment for LDH assessment. Cell death is reported as % cytotoxicity relative to a 100 % lysis control. All data is presented as a percentage of UTC control (mRNA expression/ Ribogreen / UTC) and expressed as mean ± S.E.M. Experiments were performed in triplicate with three biological replicates.

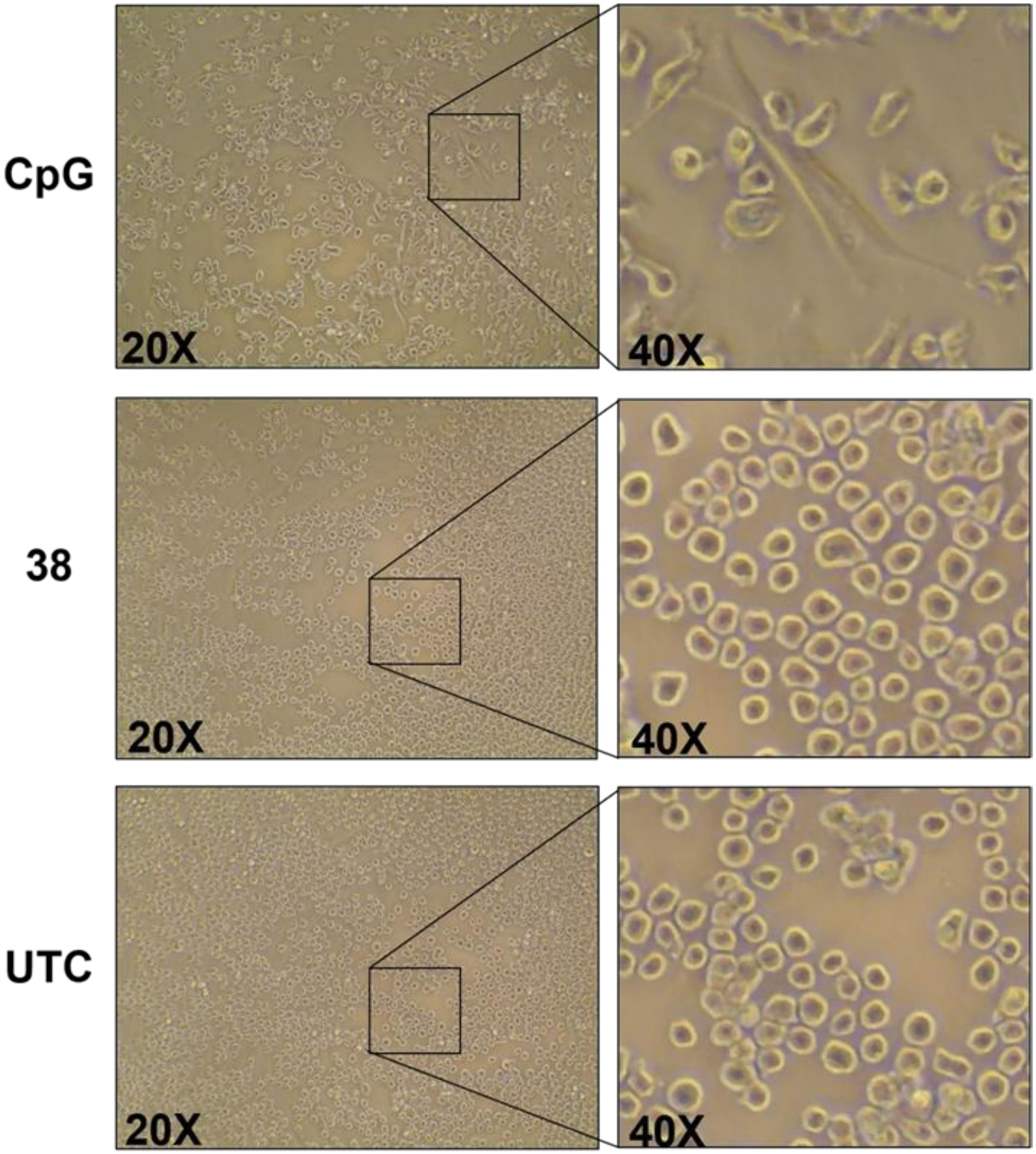

Supplementary Figure 3. Cellular morphology observed in THP1-TLR9 cells treated with 40uM of indicated PS-ASO for 2 hours. Images were taken 96-hours after treatment.

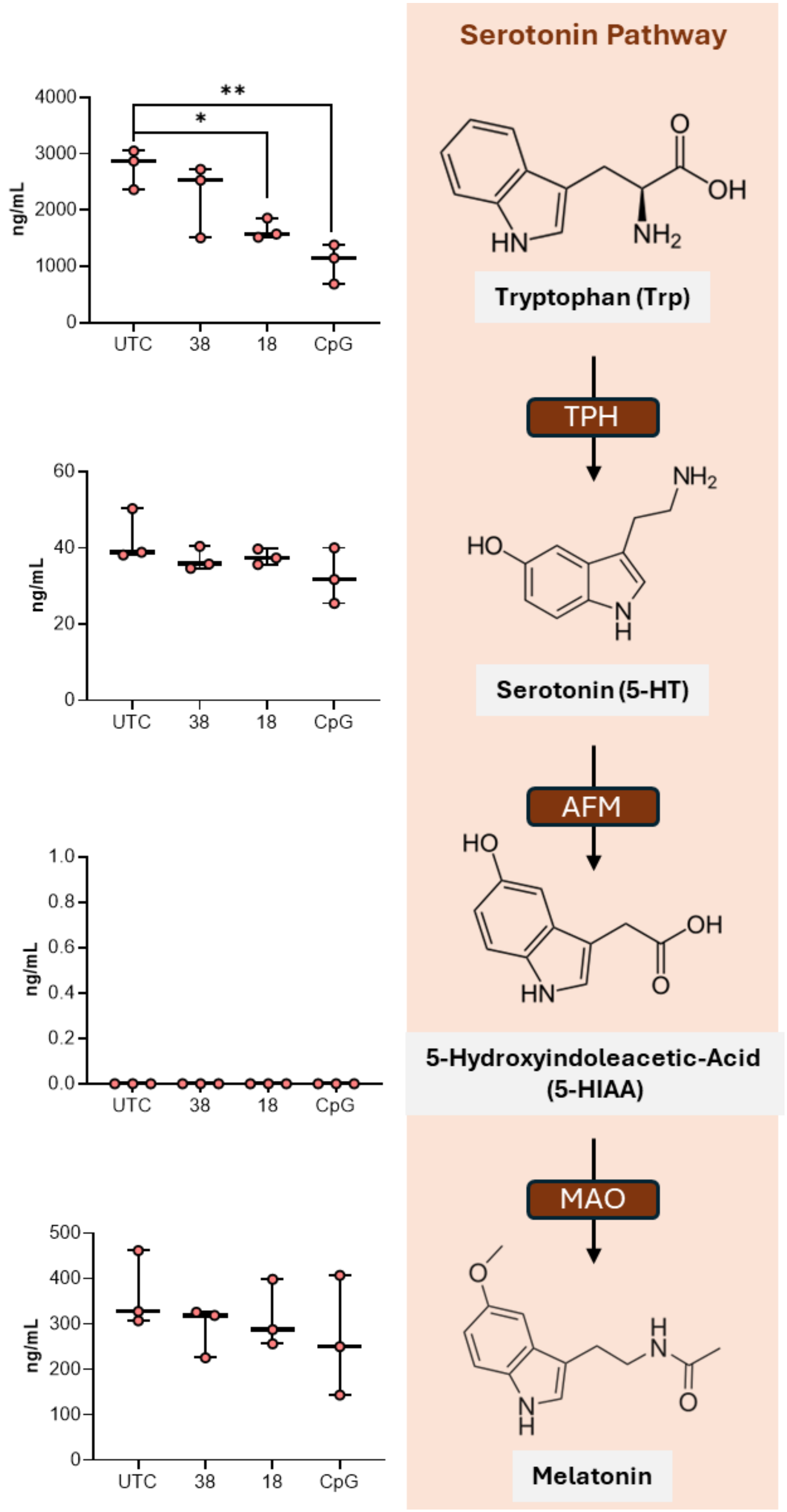

Supplementary Figure 4. Immunogenic PS-ASOs do not alter metabolite levels of the serotonin branch of tryptophan metabolism.

SUPPLEMENTARY REFERENCES:

1. Arafeh, R., Shibue, T., Dempster, J.M. *et al*. The present and future of the Cancer Dependency Map. *Nat Rev Cancer* 25, 59-73 (2025). https://doi.org/10.1038/s41568-024-00763-x
